# Similarity in Early Life Stress Exposure Is Associated With Similarity in Neural Representations in Early Adulthood

**DOI:** 10.1002/hbm.70373

**Published:** 2025-10-04

**Authors:** Miro Ilomäki, Jallu Lindblom, Marjo Flykt, Mervi Vänskä, Raija‐Leena Punamäki, Patrik Wikman

**Affiliations:** ^1^ Department of Psychology University of Helsinki Helsinki Finland; ^2^ Faculty of Social Sciences/Psychology Tampere University Tampere Finland; ^3^ Advanced Magnetic Imaging Centre, Aalto NeuroImaging Aalto University Espoo Finland

**Keywords:** early life stress, intersubject similarity, neuroimaging, procrustes, representational similarity

## Abstract

Early life stress (ELS) has profound implications for developmental trajectories, yet the neural mechanisms underlying its long‐term effects remain incompletely understood. In the present study, we examined whether interindividual similarity in ELS exposure aligns with similarity in neural representations and behavioral task performance in early adulthood. Leveraging a 20‐year longitudinal dataset of Finnish families, we evaluated 87 young adults who underwent functional magnetic resonance imaging (fMRI) during an emotional go/no‐go task. Intersubject representational similarity analysis (IS‐RSA) was used to assess the associations between pairwise similarities in prospectively or retrospectively measured ELS, neural representations in 360 cortical regions, and task performance. We incorporated multidimensional scaling and Procrustes analysis to visualize interindividual differences in neural representational spaces. Prospective ELS—but not Retrospective ELS—was significantly associated with neural representational similarity across 40 cortical regions, including the anterior insula, frontal operculum, and anterior cingulate cortex. These findings highlight the systematic and chronic effects of more moderate ELS on brain development and emphasize the value of prospective measurements and advanced similarity analyses in capturing the nuanced influences of ELS. By integrating spatial and shape analytical techniques, the present study provides new insights into the long‐term neurobiological correlates of ELS and introduces novel methodological tools for neurodevelopmental research.

## Graphical abstract

Utilizing intersubject representational similarity analysis, we present significant overlap between interindividual similarities in moderate, prospectively evaluated early life stress and cortical neural representational similarity during an emotional go/no‐go task in early adulthood. We present novel approaches to inspecting interindividual differences in neural representational spaces using multidimensional scaling and Procrustes alignment.

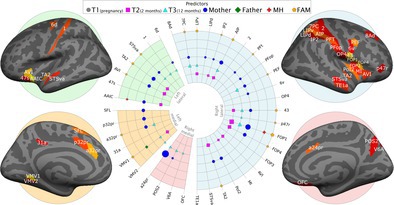

## 1 Introduction

The complex relationship between early childhood experiences and brain development is a central topic in neuroscientific research. Much of the existing literature has focused on the impact of extreme forms of early life stress (ELS), such as abuse, neglect, and maltreatment, on various developmental outcomes. Research has extensively documented the long‐term associations of extreme ELS on mental health (e.g., depression and anxiety; Baldwin et al. 2023; Gershon et al. 2013), emotion processing and self‐regulation (e.g., learning, memory, and emotion regulation; Wade et al. 2022; Pechtel and Pizzagalli 2011; Mueller et al. 2010), structural brain changes (e.g., in the amygdala and the medial prefrontal cortex; Ancelin et al. 2021; McLaughlin et al. 2019), and functional alterations (e.g., increased amygdala reactivity to negative emotional cues; Ross et al. 2021; Cohodes et al. 2020; Herzberg and Gunnar 2020; Kraaijenvanger et al. 2020; Kaiser et al. 2018). However, the effects of more moderate but chronic ELS, such as strained family relationships and parental mental health problems, are less well understood. Despite being relatively common stressors, studying their long‐term influences on brain functioning presents numerous challenges. One challenge is the common practice of categorizing participants into dichotomous “exposed” and “unexposed” groups. Such an approach may obscure the more nuanced and gradual effects of ELS on brain functioning. Additionally, many studies rely on retrospective self‐report assessments of childhood adversities. While retrospective methods are efficient and often accurate, they are prone to various biases and limitations, such as childhood amnesia (Bell and Bell 2018; Reuben et al. 2016; Lalande and Bonanno 2011; Brennan et al. 2006; Hardt and Rutter 2004). In contrast, longitudinal and prospective approaches, while costly and time‐consuming, mitigate memory‐related biases and errors. As such, they provide a more reliable method to examine the effects of ELS on adult brain functioning.

An additional limitation in neurodevelopmental research is the frequently used univariate analysis of mean activity or functional connectivity of a limited number of brain regions. Advances in neuroscientific paradigms and computational analysis methods relating to understanding neural representations (Barack and Krakauer 2021; Kriegeskorte and Kievit 2013; Decharms and Zador 2000) and individual nuances in brain function are still relatively rare in the neurodevelopmental literature, especially in ELS research. Notably, a large portion of neurodevelopmental research on structural and functional changes has focused on subcortical regions, especially on the amygdala and the hippocampus, as these regions have been strongly implicated in emotion and memory‐related processes (Phelps 2004). Additionally, abundant research has focused on binodal functional connections between (limbic) subcortical regions and (largely frontal) cortical regions hypothesized to be involved in emotional experiences and self‐regulation, such as the medial frontal gyrus, orbitofrontal cortex, the insula, and the cingulate cortex (Cohodes et al. 2020). While serving a necessary role, this univariate activation (or connectivity) analysis framework offers an incomplete picture (Freund et al. 2021). A gap thus exists in our understanding of how ELS might alter the development of larger‐scale cortical representations of emotion processing and related behavior. Regardless, some neurodevelopmental studies utilizing novel computational neuroscientific methods have surfaced. For example, Saragosa‐Harris et al. (2024) used representational similarity analysis (RSA; Kriegeskorte et al. 2008) to demonstrate that greater retrospectively evaluated ELS was associated with greater similarity in neural representations of ambiguous and threatening facial expression stimuli. Equivalent similarity approaches have been utilized in general neuroscientific studies of, for example, facial feature‐related trustworthiness evaluation (e.g., Tashjian et al. 2019; FeldmanHall et al. 2018). By employing such multivariate methods that account for individual nuances in brain function, these approaches promise a shift in the neuroscientific paradigm and an elucidation of the complex outcomes of ELS that might have been missed by univariate approaches.

In the present study, we leverage a unique, 20‐year‐spanning longitudinal dataset of Finnish families to examine the influence of ELS on task‐based cortical representations in young adults. More specifically, we explore whether interindividual similarity in ELS (i.e., in terms of overall exposure) is associated with interindividual similarity in cortical neural representations of valence‐related performance in an emotional go/no‐go task that utilizes facial expression stimuli. The original go/no‐go task was developed and has been used to study cognitive control (Gratton et al. 2018; Schulz et al. 2007). The emotional go/no‐go is an adaptation of the task that utilizes emotional stimuli, such as emotional facial expressions, that interfere with cognitive control and is thus used to assess implicit emotion regulation (Ahmed et al. 2015). Importantly, previous research has reported ELS‐related behavioral and neural alterations in facial emotion processing and recognition (Saarinen et al. 2021; Doretto and Scivoletto 2018; da Silva Ferreira et al. 2014), emotion regulation (Miu et al. 2022; Schweizer et al. 2016), and inhibitory control (Wade et al. 2022; Brieant et al. 2023). However, results from various studies remain mixed or inconclusive. The general findings, however, suggest that those who have experienced adversities or chronic stress during childhood display altered behavioral and neural responses to tasks requiring facial emotion processing, emotion regulation, and cognitive control.

To elucidate this evasive area of inquiry, we utilize RSA in conjunction with intersubject representational similarity analysis (IS‐RSA) to gauge whether interindividual similarity in ELS maps onto interindividual similarity in the neural representation of the emotional go/no‐go task. IS‐RSA is a non‐parametric computational analysis approach that essentially allows for an exploration of the association between interindividual, or pairwise, similarities in almost any variables of interest and has been increasingly utilized during the current decade (e.g., Sheng et al. 2023; Hsiao et al. 2024; Ilomäki et al. 2022; Finn et al. 2020; Rhoads et al. 2020). In IS‐RSA, measures of interindividual similarities rely on a choice of a distance metric (e.g., Euclidean, correlation, or cosine) that denotes pairwise distances for every unique dyad. In the current study, we utilize a Nearest Neighbor (NN) model, where interindividual similarity in ELS is calculated based on Euclidean distance or the arithmetic absolute pairwise difference. The NN model is, however, uninformative about the level of stress of the pair (two pairs can have the same dissimilarity value even if one pair has high overall ELS and the other has low overall ELS). The NN model is contrasted with the Anna Karenina (AnnaK) model in which the pairwise inspection involves calculating the arithmetic mean of the dyad, retaining information about the pair's absolute level of ELS. However, an extended discussion regarding the choice of an approach to and metric for pairwise distances in IS‐RSA is beyond the scope of the current paper; please see Finn et al. (2020) for a detailed outline of the topic. For comparative purposes, we also employ more commonly used linear models, complementary pairwise distance approaches (such as the AnnaK model), and utilize both prospectively and retrospectively assessed ELS. It is noteworthy that in the current study, the distinction between the assessments is not only temporal but also qualitative: Prospective ELS denotes mental health symptoms and relationship problems reported by both parents during pregnancy and infancy. Retrospective ELS denotes participant self‐reports of adverse traumatic and stress‐provoking experiences (e.g., physical and verbal abuse or bullying) that align more closely with how ELS has typically been operationalized in the literature. By leveraging these methods and the unique dataset, we aim to demonstrate how similarity in ELS is empirically mirrored in similarity in complex cortical neural representations of social–emotional information and related behavior even in early adulthood.

The present study aims to answer the following questions: (1) does Prospective or Retrospective ELS influence task performance in the emotional go/no‐go task as measured by response sensitivity, bias, and reaction times, and (2) are individuals with similar Prospective or Retrospective ELS also more similar in terms of their cortical neural representations of the task? Our approach also attempts to highlight how different operationalizations (prospective and retrospective) of ELS and different pairwise distance approaches might contribute to varying results, and to assess the contribution of the similarity analysis approach for neurodevelopmental research.

## 2 Material and Methods

### 2.1 Participants

The current study is a segment of a Finnish longitudinal research project known as Miracles of Development (MIDE). In the project, 953 Finnish families have been followed since pregnancy. Approximately half of these families conceived their child via successful assisted reproductive treatment [ART: *n* = 484 (51%); naturally conceived (NC): *n* = 469 (49%)]. During pregnancy, the inclusion criteria for the parents included being Finnish‐speaking, and additionally for the NC group, having no history of infertility and the mother's age being over 25 years to match the higher age of ART mothers. See for example, Vänskä et al. (2011) or Flykt et al. (2021) for a more detailed description of the larger study sample.

To ensure comprehensive representation of the study sample, disproportionate stratified sampling (Parsons 2017) was used in the selection of the fMRI study participants. The sampling was based on the Prospective ELS index, which included 20 variables indicating maternal and paternal mental health and family relationship problems during the pregnancy (2nd trimester) and when the child was 2 and 12 months old. See section “Prospective ELS operationalization” for a more detailed definition of the Prospective ELS index. The original MIDE sample was divided into four equal strata using this index (*z* < −0.42 for low; −0.42 ≤ *z* < 0.24 for moderate‐low; 0.24 ≤ *z* < 0.90 for moderate‐high; *z* ≥ 0.90 for high). We planned to select 24 participants from each of the four strata with a balanced selection for the child's sex and parents' infertility history. Only cases with a maximum of 8 missing values in the ELS index were considered eligible. In total, 92 participants were successfully sampled, with some deviations from the original plan (e.g., some cells were depleted and replaced by nearby cells). The sample represented all strata (*χ* ^2^ (3) = 0.35, *p* = 0.951) and was balanced for the child's sex (*χ* ^2^ (3) = 1.55, *p* = 0.671) and parents' fertility history (*χ* ^2^ (3) = 0.21, *p* = 0.976) within each stratum.

For the present neuroimaging substudy, both resting‐state and task‐based fMRI data were obtained from 92 young adults aged 18–21 years (M = 19.06, SD = 0.77; 55% female). Inclusion criteria for the fMRI study participation were right‐handedness, being a native Finnish speaker, normal hearing, normal or corrected vision, and having no current psychiatric or neurological diagnoses. Due to excessive head motion (> 0.2 mm mean framewise displacement) during the fMRI procedure, five participants were excluded from further analysis, leaving a final sample of 87 young adults for the current analyses. The fMRI experiment, along with the previous stages of the study, received approvals from the Ethics Committee of the Hospital District of Helsinki and Uusimaa, Finland.

### 2.2 Prospective ELS Operationalization

Prospective ELS was indexed over three timepoints (during pregnancy, T1; when the child was 2 months old, T2; and when the child was 12 months old, T3) through questionnaires regarding different problem domains (mental health symptoms and family relationship problems) completed by both the mother and the father.

Mental health symptoms in the family were measured using the General Health Questionnaire (GHQ36; mothers: *α* = 0.91–0.94, fathers: *α* = 0.92–0.94) (Goldberg and Hillier 1979) and the Beck Depression Inventory (BDI‐13; mothers: *α* = 0.75–0.84, fathers: *α* = 0.80–0.83) (Beck et al. 1961) at all three time points. GHQ assesses depression, anxiety, insomnia, and social dysfunction, whereas BDI assesses depression symptoms only.

Family relationship problems were evaluated using the Dyadic Adjustment Scale (DAS; mothers: *α* = 0.92–0.93, fathers: *α* = 0.91–0.91) (Spanier 1976) and the Parenting Stress Index (PSI‐36; mothers: *α* = 0.90–0.90, fathers: *α* = 0.91–0.91) (Abidin 1997) when the child was 2 and 12 months old, but not during pregnancy. The DAS measures conflicts and low affection between parents, and the PSI assesses parenting distress and difficulties in parent–child relationships.

Complete Prospective ELS data, comprising 20 variables, were available for 84% (*n* = 77) of the participants, with 9% (*n* = 8) missing eight variables, and 7% (*n* = 7) missing one to four variables. Expectation–Maximization (EM) imputation, using data from the larger study sample, was employed to handle missing data. To create a total Prospective ELS score, a cumulative risk score (see Ettekal et al. 2019) was calculated by averaging the scores from each questionnaire over time and between parents, then standardizing and averaging these scores (M = 0.00, SD = 0.86, range = −1.5–2.34). The variables had moderately high internal consistency (*α* = 0.88). See Supporting Information: Figure S1 for histograms of individual Prospective ELS items, and Figure S2 for correlations between the individual Prospective ELS item and the overall Prospective ELS score.

### 2.3 Retrospective ELS Operationalization

Retrospective ELS was assessed using the self‐report questionnaire items adapted from the Revised Adverse Childhood Experiences questionnaire (Finkelhor et al. 2015) approximately a year prior to fMRI data collection when the participants were 17–19 years old (M = 18.23, SD = 0.34).

Two items with a three‐point Likert scale (0 = *never*, 1 = *sometimes*, 2 = *often*) assessed the following: Emotional abuse (e.g., “Did a parent or other adult in the household … swear at, insult, or put you down?”); Physical abuse (e.g., “Did a parent or other adult in the household … push, grab, shove, or slap you?”); Emotional neglect (e.g., “Did you … feel that no one in your family loved you or thought you were important or special?”); and Parent treated violently (e.g., “Was your parent … pushed, grabbed, slapped, or had something thrown at her/him?”). Two additional items were added to capture common ELS events for interparental psychological violence (e.g., “Have you seen your parent being threatened by violence at home?”; Ellonen et al. 2008).

Binary response items (0 = *no*, 2 = *yes*) assessed the following: Family alcohol and drug problems (“Did you live with anyone who was a problem drinker or alcoholic, or who used street drugs?”); Peer victimization (“Have you been bullied in school?”); Parents' divorce (“Did your parents separate/divorce?”); Family mental illness (“Was a household member mentally ill?”); Death of a close person (“Have you ever lost anyone close to you by death?”); Family somatic illness (“Has any family member had a serious illness during your life?”); and Other serious adversities (“Have you experienced other adversities, such as accidents, victimization, or natural catastrophes?”). The total Retrospective ELS score was calculated by averaging the two‐item domains and then summing the scores of the 12 domains (M = 4.45, SD = 2.60; range = 0–11.50). In the current study sample, Retrospective ELS and the overall Prospective ELS are not correlated (*r* = 0.001, *p* = 0.96; see Supporting Information: Figure S3).

### 2.4 Task Description and Stimuli

**FIGURE 1:**
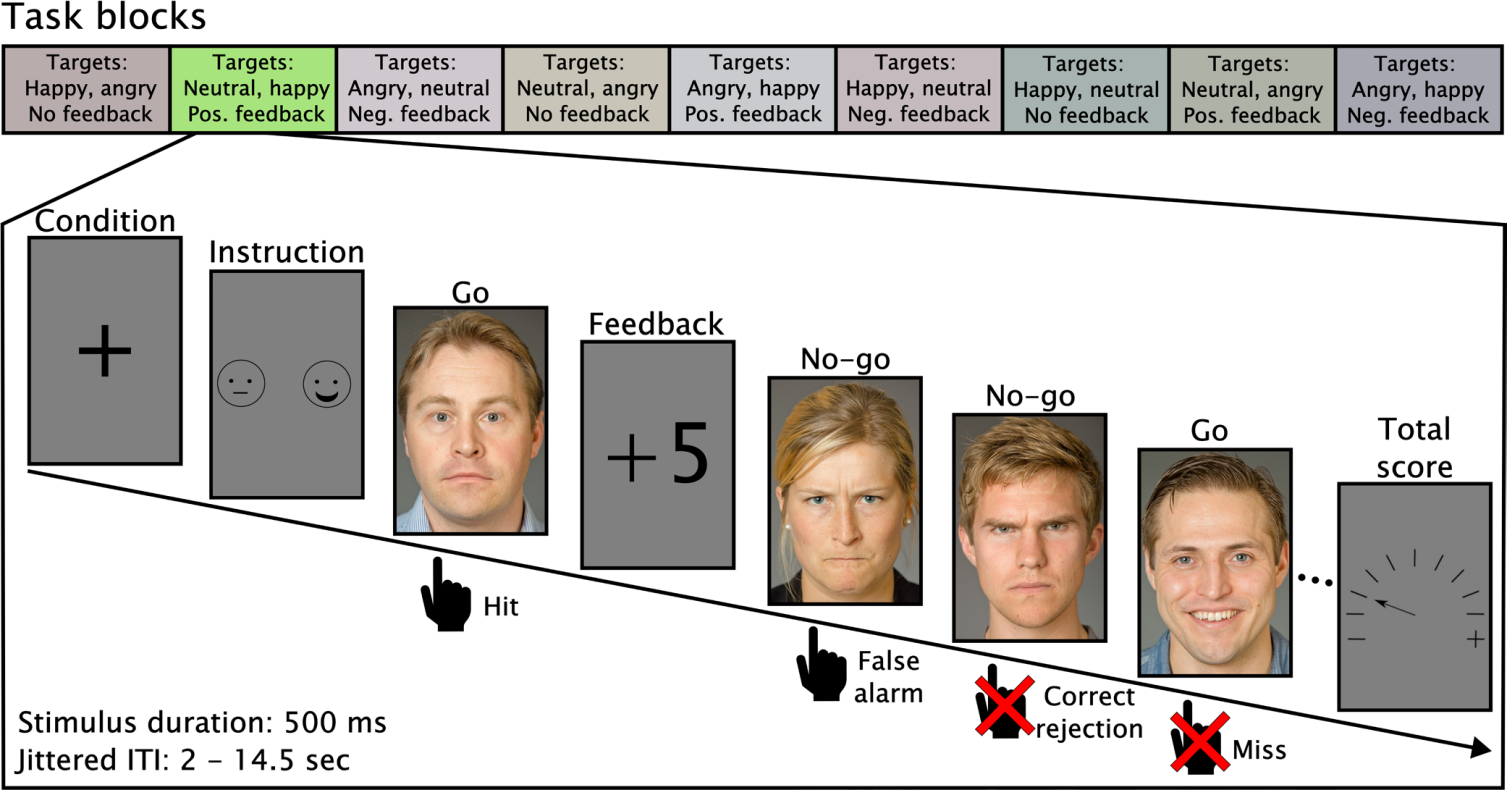
Schematic illustration of the emotional go/no‐go task. Displayed on top are the nine variants of the task blocks (control task excluded). Blocks were pseudorandomized and varied based on the target stimuli and the feedback condition. A task block with neutral and happy emotional expression stimuli as targets and angry facial expressions as non‐targets is highlighted as an example. Participants were instructed to press a button as fast as possible when a target facial expression appeared, and, depending on the task block, points were given, taken, or not adjusted after responses. Each block began with instructions about the feedback condition and the target facial expressions. Participant responses were categorized according to signal detection theory: hits and correct rejections for correct commission and omission, respectively, and false alarms and misses for commission and omission errors, respectively. The total score was always displayed at the end of each block.

Participants completed an emotional go/no‐go task (Hare et al. 2008) while undergoing fMRI (Figure 1). In this task, participants were instructed to press a button as fast as possible when a target emotional facial expression (neutral, happy, or angry; henceforth referred to as *valence*) appeared on the screen (“go” condition) and to withhold their response when a non‐target facial expression appeared (“no‐go” condition). A total of 10 blocks were performed, each consisting of 48 trials: 32 target trials and 16 non‐target trials. One of these blocks was a control task. Stimuli consisted of photographs of three facial expressions (happy, angry, and neutral) of 16 different identities from the Umeå University Database of Facial Expressions (Samuelsson et al. 2012). Each stimulus was presented for 500 ms, followed by a jittered intertrial interval ranging from 2 to 14.5 s. Each task block comprised a specific pairing of two targets that were instructed before the start of each block, with blocks pseudorandomized across blocks (e.g., happy–angry, happy–neutral, or angry–neutral). Each task block was also accompanied by one of three types of feedback conditions (no feedback, positive feedback, and negative feedback). In one‐third of the blocks, points were awarded for correct trial responses (positive feedback). In one‐third, points were deducted for incorrect responses (negative feedback). In one‐third, no feedback was given. The feedback condition was instructed before the instruction for the target facial expressions (a dot for no feedback, “+” for positive feedback, and “‐” for negative feedback). Feedback was given by presenting text on the screen, for example, “+5”. Positive feedback was given on random trials with correct responses (on average 5 times over all correct responses within a positive feedback block). Positive feedback was an addition of five points to the participant's total score. However, approximately 1 out of every 5 positive feedback presentations was a larger number of points (“+30” points). Negative feedback was always a subtraction of five points and was always given after an incorrect response in the negative feedback blocks. To make the blocks with no feedback as visually similar as the blocks with feedback, random presentations of *Xs* with the same number of tokens as in the feedback conditions were presented in a similar manner. Whenever feedback was displayed in a trial, it was presented immediately after the response. The total score was visually represented using a semicircular gauge, akin to a car speedometer or compass. The display consisted of a half‐circle, with an arrow that indicated the score by pointing to specific positions along the semicircle. The leftmost side of the half‐circle represented the lowest possible score, and the arrow moved clockwise as the score increased, with the rightmost end representing the highest possible score. This allowed participants to quickly grasp their performance without relying on numerical values. There were short breaks between blocks, approximately 15 s, during which participants received instructions for the feedback condition and the targets for the next block. During this time, participants viewed a fixation cross before the next block of trials began. There was an additional control task block, during which participants were presented with the same facial expression stimuli under the same timing conditions but were instructed to respond to every stimulus. No feedback was given during the control block (analyses pertaining to this task were not included in the manuscript).

### 2.5 Behavioral Task Performance

The primary dependent variables of the behavioral analyses were *d*‐prime (*d′*), criterion (*c*), and reaction times (RT) to hits (responses to target stimuli). *D*‐prime is a metric for signal detection sensitivity, while *c* is a metric for bias, or the tendency to favor one type of response over another. Higher values of *d′* indicate greater ability to distinguish target stimuli from non‐target stimuli, while higher absolute values of c reflect a stronger tendency toward one type of response. To calculate *d′*, the *z*‐transformed false alarm (FA) rate was subtracted from the *z*‐transformed hit rate (HR): *d′* = *z*(HR)−z(FA), while the criterion was calculated by taking the average of the z‐transformed HR and FA and multiplying it by −1: $c=-\left(\frac{z\left(\mathrm{HR}\right)+z\left(\mathrm{FA}\right)}{2}\right)$.

Linear mixed‐effects models were employed to analyze behavioral task performance metrics. RT, *d′* and *c* were modeled separately to investigate the effects of ELS and other covariates on task performance. Separate models were also run for Prospective and Retrospective ELS. ELS, valence (neutral, angry, and happy), mother's age, mother's socioeconomic status (SES; indexed as mother's education using 5‐level grading from 0 = *university level education* to 4 = *no vocational education*), participant sex, and ART were included as fixed effects variables. Subject ID was specified as a random intercept and valence was allowed to vary within subject by including a random slope for valence. Interaction effects were modeled for ELS × valence, ELS × participant sex, ELS × mother's age, ELS × mother's SES, and ELS × ART‐status. Likelihood ratio tests (LRTs) were used to compare the fit of models with and without ELS predictors to determine the added explanatory power of ELS. *F*‐statistics with Satterthwaite approximations were used to evaluate the significance of main effects and interaction terms within the linear mixed‐effects framework. RT data were log‐transformed to correct for skewness prior to analysis. Additionally, *d′* was used in its respective similarity analyses (see “Complementary similarity analyses” results section). The behavioral analyses were conducted in *R* using the lme4 package for mixed‐effects modeling.

### 2.6 fMRI Data

#### 2.6.1 Acquisition

All data were acquired using a 3 T MAGNETOM Skyra whole‐body scanner (Siemens Healthcare, Erlangen, Germany) with a 20‐channel head coil at the Advanced Magnetic Imaging (AMI) Centre, Aalto NeuroImaging, Aalto University School of Science, Espoo, Finland. During the imaging procedure, resting‐state, structural, and task‐based data were collected. The first task in the scanning session was the go/no‐go task. The second task was a Reading the Mind in the Eyes task (RMET), involving photographs of faces cropped to show eyes only (adapted from Moor et al. 2012), where participants had to pick a word that either describes (1) the thoughts or feelings of the person in the photograph, or (2) the gender and age of the person in the photograph. Next, an anatomical scan [MPRAGE; high‐resolution 3D T1 anatomical images (voxel matrix 256 × 256, in‐plane resolution 1 mm × 1 mm × 1 mm)] was obtained. These anatomical images were used in the current study. Next, participants completed a social media task in which they posted opinions to a bogus Facebook group created by the experimenters and received peer feedback on those opinions (see Wikman et al. 2022). Finally, resting state data (participants were asked to lie still with eyes open) were acquired. All functional runs used echo‐planar imaging (EPI) with an imaging area covering the whole brain comprising 43 contiguous oblique slices (TR 2500 ms, TE 32 ms, flip angle 75°, voxel matrix 64 × 64, field of view 20 cm, slice thickness 3.0 mm, in‐plane resolution 3.1 mm × 3.1 mm × 3.0 mm). Participants were reimbursed 15 €/h (2–3 h) for their time.

#### 2.6.2 Preprocessing

We used fMRIPrep 20.2.5 (Esteban, Blair, et al. 2018; Esteban, Markiewicz, et al. 2018) to preprocess the functional and structural MRI data. The anatomical T1‐weighted (T1w) image was corrected for intensity non‐uniformity (INU) with N4 Bias Field Correction (Tustison et al. 2010), distributed with ANTs 2.3.3 (Avants et al. 2008), and skull‐stripped with a Nipype implementation of the antsBrainExtraction.sh workflow (from ANTs), using OASIS30ANTs as the target template. Brain tissue segmentation of cerebrospinal fluid (CSF), white matter (WM), and gray matter (GM) was performed on the brain‐extracted T1w using fast (FSL 5.0.9, Zhang et al. 2001). Spatial normalization to standard space (MNI152NLin6Asym) was performed through nonlinear registration with antsRegistration (ANTs 2.3.3), using brain‐extracted versions of both the T1w reference and the T1w template. The following preprocessing was performed for the fMRI data: First, a reference volume and its skull‐stripped version were generated using a custom methodology of fMRIPrep. A deformation field to correct for susceptibility distortions was estimated based on fMRIPrep's fieldmap less approach. The deformation field is that resulting from co‐registering the BOLD reference to the same‐participant T1w reference with its intensity inverted (Huntenburg 2014; Wang et al. 2017). Registration is performed with antsRegistration (ANTs 2.3.3), and the process was regularized by constraining deformation to be nonzero only along the phase‐encoding direction and modulated with an average fieldmap template (Treiber et al. 2016). Based on the estimated susceptibility distortion, a corrected EPI reference was calculated for a more accurate co‐registration with the anatomical reference. The BOLD reference was then co‐registered to the T1w reference using bbregister (FreeSurfer), which implements boundary‐based registration (Greve and Fischl 2009). Co‐registration was configured with 6° of freedom. Head‐motion parameters with respect to the BOLD reference (transformation matrices and six corresponding rotation and translation parameters) are estimated before any spatiotemporal filtering using mcflirt (FSL 5.0.9, Jenkinson et al. 2002). BOLD runs were slice‐time corrected using 3dTshift from AFNI 20160207 (Cox and Hyde 1997) and resampled onto their original, native space by applying a single, composite transform to correct for head motion and susceptibility distortions. The BOLD time series were then resampled into standard space (MNI152NLin6Asym). Confounding time series for framewise displacement (FD), DVARS, and three region‐wise global signals were calculated based on the preprocessed BOLD. FD was computed using two formulations following Power (absolute sum of relative motions, Power et al. 2014) and Jenkinson (relative root mean square displacement between affines, Jenkinson et al. 2002). FD and DVARS are calculated for each functional run, both using their implementations in Nipype (following the definitions by Power et al. 2014). The three global signals are extracted within the cerebrospinal fluid (CSF), the white matter (WM), and the whole‐brain masks (global signal, GS). Additionally, a set of physiological regressors were extracted to allow for component‐based noise correction (aCompCor, Behzadi et al. 2007). Gridded (volumetric) resamplings were performed in a single interpolation step using antsApplyTransforms (ANTs), configured with Lanczos interpolation to minimize the smoothing effects of other kernels (Lanczos 1964).

#### 2.6.3 First Level Model

We used FEAT (FMRI Expert Analysis Tool, version 6.00), part of FSL (FMRIB's Software Library; www.fmrib.ox.ac.uk/fsl), to perform two separate first‐level general linear model (GLM) analyses. Altogether, possible estimatable variables comprised combinations of factors Valence (neutral, happy, and angry), Correctness (correct and incorrect), Motor response (response and no‐response), and Feedback (no‐feedback, positive feedback, and negative feedback). However, because these conditions depended on the behavior of the participant, it was impossible to estimate all the combinations of the conditions for each participant. Therefore, we opted to run two separate GLMs: the first model included factors Valence (neutral, happy, and angry), Correctness (correct and incorrect), and Motor response (response and no‐response), henceforth referred to as the *performance model* (12 regressors of interest). Importantly, the factoring into Correctness and Motor response is equivalent to the signal detection metrics (hit = response + correct; miss = no‐response + incorrect; false alarm = response + incorrect; correct rejection = no‐response + correct). In the other model, we included Feedback as a factor, and instead of the factors Correctness and Motor response, a factor for Target (target and non‐target) was employed. Thus, this model included the factors Target (target and non‐target), Valence (neutral, happy, and angry), and Feedback (no‐feedback, positive feedback, and negative feedback), henceforth referred to as the *reward model* (18 regressors of interest). Note that the reward model was only used in this study to define regions of interest for a specific similarity analysis (see “Complementary similarity analyses”). In both models, we added regressors for all the control tasks, the feedback presentation, and the presentation of the total score (which were not analyzed in this manuscript). In all GLMs, regressors were included in FSL's three‐column format, including their temporal derivatives (start time in seconds, trial duration 1 s). To account for motion, drift, and other nuisance factors, the model included the following regressors calculated by *fMRIPrep*: GS, the six basic motion, and the discrete cosine basis functions. A smoothing of 5 mm full width half maximum (FWHM) and whitening was applied in FEAT when running the model.

### 2.7 Second Level Analyses

For univariate within‐subjects analysis, we ran a repeated measures ANOVA model across the whole functional volume using the Multivariate Repeated Measures (MRM) toolbox in Matlab (McFarquhar et al. 2016). The within‐subjects factors for the model were Valence (neutral, happy, and angry), Motor response (response vs. no response), and Correctness (correct response vs. incorrect response). We assessed all main effects and interactions of these factors.

We additionally ran two whole‐brain ANCOVAs including the same within‐subjects factors specified above and the between participants variables: Prospective and Retrospective ELS (separately in their respective models), with mother's age and SES, participant sex, and ART included as zero‐weighted covariates to isolate their influence.

In all second‐level models, we applied a cluster‐based thresholding approach using permutation testing with 1000 iterations in each model to address multiple comparisons. We defined clusters at an uncorrected *p* value threshold of 0.001. To control for Type I errors, we implemented a cluster‐level family‐wise error rate (FWER) correction with a threshold of 0.05.

### 2.8 Interindividual Similarity Analyses

We utilized IS‐RSA to assess the associations between ELS, neural representations, and behavioral task performance. IS‐RSA is a non‐parametric method that examines relationships between variables by comparing interindividual similarities constructed across the sample. The analysis involves computing pairwise distances for each variable of interest, followed by comparing these distances across the variables. Specifically, we generated intersubject dissimilarity matrices (ISDMs) for each variable of interest, where each matrix denotes pairwise comparisons between all unique pairs of participants, quantified using a distance metric tailored to the variable type. For single‐score aggregates or indices, such as the Prospective and Retrospective ELS scores, we employed Euclidean distance, which is equivalent to the absolute arithmetic difference in the pairs' ELS scores. For multidimensional vectors comprising the neural representational profiles, we used correlation distance (calculated as 1−*r*) to capture structural differences in the data rather than raw value differences.

**FIGURE 2:**
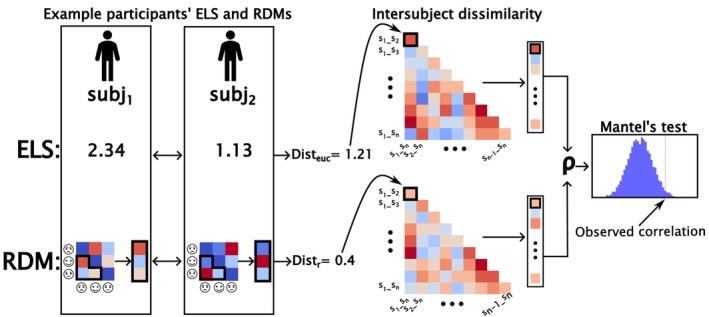
Schematic of the analysis steps for IS‐RSA using the ELS ISDM versus ROI ISDM analysis for one brain region as an example. Reading from left to right, first, each participant pair's ELS and RDM are compared. The ELS is compared with simple Euclidean distance (*dist*_*euc*_), whereas the RDMs are compared by first vectorizing the non‐redundant portion of the RDM, and then using correlation distance (*dist*_*r*_) to attain a dissimilarity value for the pair. Finally, all pairwise dissimilarities are placed in their respective positions in the intersubject dissimilarity matrix. The matrix comprises all the unique pairwise distances attainable from the current sample, spanning from comparing *subj*_1_ to *subj*_2_, to comparing *subj*_*n*−1_ to *subj*_*n*_. Then, the ELS and ROI intersubject dissimilarity matrices are vectorized, and a partial Spearman correlation is calculated between them, with mother's age and SES, participant sex, and ART status partialed out. This attained correlation is finally tested by comparing it to the distribution of resampled correlations. These resampled correlations are attained by shuffling the rows and columns of one of the matrices, vectorizing it, recalculating the correlation, and repeating this process thousands of times. If the observed correlation deviates enough from the expected correlation based on the resampling, it will be deemed statistically significant.

To establish an effect size for the comparisons between the ISDMs, the following steps were taken: the redundant upper triangle of each ISDM was removed, alongside the diagonal (self‐self‐comparisons that are always maximally similar), resulting in all possible unique pairwise comparisons. In our sample, this resulted in $\frac{87\;\left(87-1\right)}{2}=3741$ unique pairwise comparisons for each ISDM. Finally, the ISDMs were vectorized and then correlated using partial Spearman correlation to establish the monotonic relationship between the rank orders of the pairwise distances, while controlling for confounding variables (mother's age and SES, participant sex, and ART‐status). See Figure 2 for a schematic illustration of the general analysis steps.

For neural representations of the task, we computed representational dissimilarity matrices (RDMs; Kriegeskorte et al. 2008) for every cortical Region of Interest (ROI) for each participant. ROIs were defined using the Human Connectome Project multimodal parcellation 1.0 (HCPMMP1.0; Glasser et al. 2016). The HCPMMP1.0 parcels the brain into 360 modal‐specific regions with 180 ROIs per hemisphere. Each RDM comprised comparisons between a total of 12 conditions consisting of all the combinations of emotional expression type (neutral, happy, and angry) and signal detection theory‐derived response type (hit, correct rejection, false alarm, and miss). To compute the dissimilarities for the elements of the RDM for any given region, we calculated the correlation distances between all the unique pairs of the 12 conditions (e.g., correlation distance between happy‐hit and angry‐false alarm) using vectorized condition‐related mean percent signal changes of every voxel in the region. Finally, we computed the ISDMs separately for each region by calculating the correlation distance for each unique participant pairwise comparison using their vectorized RDMs after removal of the redundant upper triangle and the diagonal of the RDM.

Due to the inflation of the amount of datapoints and the potential non‐normality in the pairwise comparisons data, Mantel's test was used to establish *p* values for the ISDM correlations. In the Mantel's test, the rows and columns of one of the compared matrices are shuffled and the correlation is recalculated. This procedure is then repeated thousands of times, and the observed correlation is compared to the distribution of correlations attained after reshuffling. For a positive one‐tailed test where pairwise similarity in *X* is associated with pairwise similarity in *Y*, the *p* value is then the amount of correlation values higher than the observed correlation divided by the number of reshufflings (or 1−percentile of the observed correlation within the distribution of the permuted correlations). The Mantel's test was performed for 360 ISDM correlations (once for each region from the HCPMMP 1.0 parcellation) for both Prospective and Retrospective ELS separately, each with 10,000 permutations. Here, False discovery rate (FDR) was used for correction of the family‐wise error rate due to multiple comparisons.

### 2.9 Visualizing Neural Representational Spaces as a Function of Prospective ELS

**FIGURE 3:**
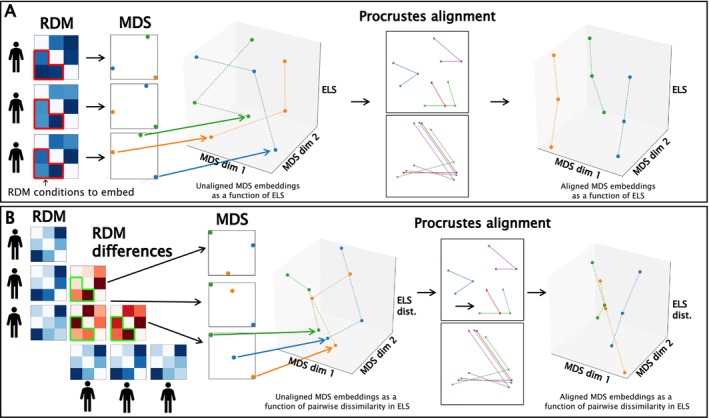
Schematic illustration of the general process for Procrustes Analysis and multidimensional scaling (MDS). First, (A) the raw RDM values, or (B) the pairwise differences in RDM values, are embedded into two‐dimensional multidimensional spaces. Because the different multidimensional spaces are not properly aligned about their dimensions, Procrustes analysis is used to align these spaces as closely as possible by matching the spaces to a reference, or in the Generalized Procrustes Analysis (GPA), into a consensus space comprising all spaces. The multidimensional space embeddings, where each point within the embedding represents a condition (e.g., happy‐hit, represented in the figure through the coloring of the points in the MDS space) in the RDM (12 points total for each MDS), are then stacked based on either (A) the raw overall Prospective ELS or (B) pairwise differences in Prospective ELS. In the current study, regions belonging to the same cluster were first MDS embedded and then aligned within a participant or participant pair (depending on whether visualizing changes in raw RDMs or pairwise differences in RDMs) using GPA over all regions within the cluster, after which GPA was used again to align the spaces over participants or pairs of participants (i.e., as a function of Prospective ELS or pairwise differences in Prospective ELS). Due to the noisiness of the data, 3‐ and 5‐degree polynomials were fit (respectively for raw RDMs and pairwise differences in RDMs) through each RDM condition specific (e.g., happy‐hit) set of points, resulting in 12 curves in one plot. The resulting images denote (A) how the RDM values change as a function of Prospective ELS and (B) how pairwise dissimilarities in the RDM elements change as a function of pairwise dissimilarity in Prospective ELS.

Due to the Nearest Neighbor model, the IS‐RSA result is relatively uninformative about what underlies the observed correlations. Thus, after attaining the IS‐RSA results, we utilized several novel approaches to verify and inspect the results. To this end, we calculated the correlations between the pairwise distances in each significant ROI's RDM element and the pairwise distance in ELS. This was achieved by subtracting each participant pair's RDMs from each other, resulting in *difference RDMs* where each element of the resulting matrix denotes the absolute difference between the values of that element for each pair of participants. This difference RDM was then used for calculating Spearman's correlations between the pair's difference in Prospective ELS and the element‐wise absolute differences of the RDMs. This resulted in a matrix for each significant ROI from the Prospective ELS ISDM versus ROI ISDM analysis that is informative about the direction and consistency of pairwise change in each element of the ROI‐specific RDM as a function of pairwise change in Prospective ELS. Next, we utilized both multidimensional scaling (MDS; Mead 1992) and Procrustes analysis by applying two‐dimensional MDS on these matrices. Procrustes analysis, first introduced by Schönemann (1966), is a low‐complexity approach to shape correspondence utilized in various scientific fields, including neuroscience (Guntupalli et al. 2016; Haxby et al. 2011). Because the MDS applied to these matrices results in a variety of two‐dimensional spaces that cannot be directly compared due to their deviations in underlying spatial alignment, Procrustes analysis is utilized to find a common two‐dimensional space. Procrustes analysis takes in a set of points or vectors (also called the *reference*) and applies the optimal transform (scaling, rotations, and reflections) to a second set to minimize the sum of the squares of the point‐wise differences between the reference and the second set. In the Generalized Procrustes Analysis (GPA; Gower 1975), instead of aligning two shapes, all instances of shapes are superimposed onto the reference shape, yielding a mean shape that is then used as the reference. After calculating the matrices denoting correlations between pairwise differences in the RDM cells and pairwise differences in Prospective ELS for each significant ROI, these matrices were then transformed into correlation distance matrices (by subtracting each element's value from 1) and projected onto two‐dimensional MDS spaces of their own. These MDS spaces were then aligned using Procrustes analysis, yielding a ROI‐by‐ROI matrix denoting Procrustes disparities (sums of the squared differences between two sets of shapes) between each ROI pair, akin to a distance matrix. Next, we utilized Ward's method (Ward 1963) for hierarchical clustering of this ROI‐by‐ROI matrix, resulting in four distinct clusters (silhouette score = 0.647). See Figure 3 for a schematic depicting the general steps for MDS and Procrustes analysis. See Supporting Information for visualizations of the element‐wise mean and standard deviation values of the correlations between the difference RDMs and Prospective ELS dissimilarities (Figure S4), and the ROI‐by‐ROI Procrustes disparity matrix with the surface projected ROIs of each cluster (Figure S5).

Finally, within each cluster, we visualized both (1) how the RDM elements changed as a function of Prospective ELS, and (2) how pairwise differences in the RDM elements changed as a function of pairwise differences in Prospective ELS. Here, Generalized Procrustes Analysis was utilized by taking for each participant, or participant pair, all the ROI‐specific RDMs, or pairwise differences in the ROI‐specific RDMs, projecting them onto two‐dimensional MDS space, and aligning them to find a “consensus space” (akin to the average MDS configuration over ROIs within a cluster). We then utilized Generalized Procrustes Analysis again, this time over participants, or pairs of participants, within the cluster to map how these consensus spaces evolved as a function of Prospective ELS, or pairwise differences in Prospective ELS. Due to the noisiness of these data, 3‐ and 5‐degree polynomials were fit through the datapoints of each of the 12 elements of the RDM among the set of consensus spaces across participants and participant pairs, respectively.

### 2.10 Complementary Similarity Analyses

To explore the main result from the Prospective ELS ISDM versus ROI ISDM analysis, individual domains of the ELS aggregate were separately tested in the same way as in the main analysis, but only for the significant regions from the main analysis. Domain‐specific ELS ISDMs were constructed separately by using the average score from each domain (responses during pregnancy, T1 [only BDI and GHQ]; when the child was 2 months old, T2; when the child was 12 months old, T3; responses from the mother only; responses from the father only; mental health‐related questionnaires, MH [BDI + GHQ]; and family relationship problems questionnaires, FAM [RDAS + PSI]). Each domain was tested with Mantel's test with 5000 permutations and FDR correction at the 0.05 alpha level.

Next, in addition to our primary Nearest Neighbor model where each dyad's predictor is the absolute difference in their total Prospective ELS score, we ran complementary IS‐RSAs using four alternative approaches for denoting pairwise distances for the Prospective ELS ISDM: (1) Euclidean pairwise distances in a 20‐dimensional space comprising each individual item of the Prospective ELS score (e.g., mother's BDI at T2, father's PSI at T3, etc.), (2) Euclidean pairwise distances in a 7‐dimensional space comprising the average ELS values from all major domains (timing: T1, T2, T3; parent: mother's responses, father's responses; problem domain: mental health related problems, family related problems), (3) Anna Karenina model, where each unique dyad's value corresponds to their average Prospective ELS, and (4) an interaction between Anna Karenina and Nearest Neighbor, where each unique dyad's value corresponds to the product of the dyad's AnnaK and NN scores.

Finally, we explored whether IS‐RSA could be used to capture between‐subjects variability in models mirroring our linear models for behavioral performance and fMRI. More specifically, we tested whether Prospective or Retrospective ELS ISDM correlated with ISDMs reflecting behavior (*d*′) and brain function during commission errors to angry faces. For behavioral data, we calculated *d*′ separately for all the levels of the two within‐subjects factors Valence (neutral, happy, and angry) and Feedback (no feedback, positive feedback, and negative feedback). Even though the feedback conditions are not specifically analyzed in the present study, we included them in the calculations of participants' *d*′ profiles to capture more idiosyncratic performance‐related variance between individuals. After calculating the *d′* profiles for each participant, we computed the *d′* ISDM by calculating the correlation distance between each unique participant pair's *d′* profiles. Regarding brain function, we ran a repeated measures ANOVA model across the whole functional volume using the Multivariate Repeated Measures (MRM) toolbox in Matlab (McFarquhar et al. 2016). We originally planned to define ROIs that were selective for faces with angry valence in the performance model (see “First level model”). However, this model yielded few ROI candidates. Therefore, we defined our ROI clusters based on the reward model with factors Target (target and non‐target), Valence (neutral, happy, and angry), and Feedback (no feedback, positive feedback, and negative feedback). After running the model, we inspected all statistically significant clusters for angry facial expression‐related effects in all contrasts including the factor Valence (main effect of Valence, Target vs. Valence; Feedback vs. Valence, and Target vs. Feedback vs. Valence) using the MRM post‐estimations tools. Seven volumetric clusters with high or low reactivity to angry facial expressions (compared to happy or neutral facial expressions and irrespective of the levels of the other factors) were then extracted and converted to binary masks. These clusters extended: (1) left amygdala, (2) left fusiform gyrus, (3) bihemispheric parahippocampal gyrus, (4) bihemispheric medial frontal cortex, (5) left inferior frontal gyrus, (6) a large bihemispheric cluster spanning the lateral and medial frontal cortex, and (7) a large right occipitoparietal cluster. Informed by previous research on the influence of ELS on facial expression processing and threat detection (Saragosa‐Harris et al. 2024; Saarinen et al. 2021; Gollier‐Briant et al. 2016; Pine et al. 2005), these clusters were then used as ROIs by extracting their average percent signal changes compared to the mean functional signal in trials where the participant committed a false alarm during an angry facial stimulus presentation (henceforth referred to as “angry FA”). Each participant thus received a profile comprising seven signal change percentage values, one for each ROI. This profile was then used to create an ISDM by calculating a correlation distance between the angry FA profiles of each unique dyad. A partial Spearman correlation was then calculated between the angry FA ISDM and Prospective ELS ISDM, Retrospective ELS ISDM, and *d′* ISDM. Overall, the following ISDM correlations were performed and tested with Mantel's test: (1) Prospective ELS ISDM versus *d′* ISDM, (2) Retrospective ELS ISDM versus *d′* ISDM, (3) Prospective ELS ISDM versus angry FA ISDM, and (4) Retrospective ELS ISDM versus angry FA ISDM. Finally, to quantify how much of the Prospective ELS ISDM versus *d′* ISDM relationship reflects covariance shared with angry FA ISDM, we fitted a simple associative path model using the Lavaan toolbox (Rosseel 2012) in Matlab. This was achieved using a similar approach as in the Mantel's test with 5000 iterations by shuffling the Prospective ELS and *d′* ISDMs, recalculating the model's z‐values, and comparing the observed z‐values to the resampled z‐values. See Supporting Information (Figure S6) depicting the seven volumetric clusters, alongside a schematic representation of the analysis.

## 3 Results

### 3.1 Behavioral Task Performance Results

Linear mixed effect models were used to investigate the association between ELS and participants' *d*‐prime (*d′*), criterion (*c*), and reaction times (log‐transformed) during the emotional go/no‐go task. Likelihood ratio tests were conducted to compare models with and without Prospective or Retrospective ELS. By‐subject random intercepts and random slopes for valence were included to account for both individual differences in overall performance and variability in response to specific emotional expression stimuli.

#### 3.1.1 *D*‐Prime

The full model significantly improved upon the null model fit when Prospective ELS and its interactions were included (LRT: ^2^(10) = 24.65, *p* = 0.006), but not when Retrospective ELS and its interactions were included (LRT: ^2^(9) = 11.014, *p* = 0.275). The Prospective ELS model revealed a statistically significant interaction between Prospective ELS and mother's age (*F*(1, 70.99) = 6.768, *p* = 0.011, *β* = 0.116) using type III Satterthwaite tests. Simple‐slope analyses at −1 SD, mean, and +1 SD of mother's age yielded Prospective ELS slopes of 0.09 (SE = 0.61, 95% CI [−1.10, 1.28]), 0.54 (SE = 0.58, [−0.60, 1.68]), and 1.00 (SE = 0.60, [−0.19, 2.18]), respectively. Although the interaction indicates that the effect of ELS on *d′* increases with maternal age, none of the conditional slopes alone reached significance. Across emotions, *d′* was highest for angry (M = 4.17), intermediate for neutral (M = 4.10), and lowest for happy (M = 3.82) facial expression stimuli (all SE≈0.23).

#### 3.1.2 Criterion

Neither of the full models incorporating Prospective ELS (LRT: ^2^(10) = 8.242, *p* = 0.605) nor Retrospective ELS (LRT: ^2^(9) = 7.874, *p* = 0.547) improved upon the null model for *c*. In the null model, both valence ((2154.29) = 13.173, *p* < 0.001) and participant sex ((1, 79.02) = 9.157, *p* = 0.003) had a significant main effect on *c*. For valence, *c* was lowest for angry (M = −1.24), slightly higher for happy (M = −1.08), and highest for neutral (M = −0.96) facial expression stimuli (all SE≈0.08). Between‐subjects, female participants displayed lower *c* (M = −1.21, SE = 0.008) than male participants (M = −0.97, SE = 0.007).

#### 3.1.3 Reaction Time

LRTs showed no improvements in model fit when adding Prospective ELS (LRT: ^2^(10) = 7.272, *p* = 0.700) or Retrospective ELS (LRT: ^2^(9) = 10.668, *p* = 0.299) in the model. No significant main effects emerged in the null model.

### 3.2 Univariate Whole Brain Results

The primary within‐subjects analysis (performance model) revealed significant main effects for the factors of Correctness (correct vs. incorrect responses), Response type (response vs. nonresponse), and Valence (neutral vs. happy vs. angry). For correct versus incorrect responses: significant clusters were observed bilaterally in the postcentral gyrus and precentral gyrus. Additional significant clusters for correct versus incorrect were detected in the insula, cuneus, middle occipital gyrus, and superior temporal gyrus, among others. For response versus nonresponse: significant clusters were found in the bilateral inferior temporal gyrus, precuneus, postcentral gyrus, and fusiform gyrus. For valence: significant clusters were observed in the right inferior frontal gyrus, the left superior temporal gyrus, and the right middle frontal gyrus. Refer to Supporting Information (Figure S7) for the surface‐projected effects.

No significant clusters were identified after FWE correction (*p* > 0.05) in neither the performance nor the reward model when incorporating between‐subjects covariates, including either Prospective or Retrospective ELS, in addition to participant sex, mother's age, and SES, and ART history.

### 3.3 Interindividual Similarity Analysis Results

#### 3.3.1 Exploratory Whole Cortex IS‐RSA Results

We utilized IS‐RSA to explore the correlations between pairwise differences in Prospective and Retrospective ELS and pairwise differences in neural representations within the 360 HCPMMP1.0 regions, while controlling for demographic covariates (see “Interindividual similarity analysis” in the Methods section for details). Prospective and Retrospective ELS were both tested against the 360 regions separately. As presented in Figure 2, the correlations were attained by computing ISDMs for all the variables of interest using either Euclidean or correlation distance and calculating a partial Spearman's correlation between the Prospective or Retrospective ELS ISDM and each of the ROI specific ISDMs, while controlling for mother's age and SES, participant sex, and ART‐status.

The 360 Prospective ELS ISDM versus ROI ISDM tests revealed significant positive correlations for 40 cortical regions after FDR correction at the 0.05 alpha level, with significant correlations ranging from *r*_*s*_ = 0.10 to *r*_*s*_ = 0.24. Out of all the Prospective ELS ISDM versus ROI ISDM correlations, 95.5% were positive, with an average correlation of *r*_*s*_ = 0.071 (*r*_*s*_‐min = −0.045; *r*_*s*_‐max = 0.245) and a median uncorrected *p* value of *p* = 0.07 (*p*‐min = 0.00001; *p*‐max = 0.87). Notable significant regions seen only in the right hemisphere included frontal opercular regions, orbitofrontal cortex, lateral intraparietal cortex, inferior frontal gyrus, dorsolateral prefrontal cortex, and inferior regions around the precentral gyrus, including the prefrontal eye field. Notable significant regions only observed in the left hemisphere included the postcentral gyrus and ventromedial visual areas. Bihemispherically, significant positive correlations were observed in regions such as the anterior insula, anterior cingulate, and anterior regions around the middle and superior temporal gyrus.

None of the Retrospective ELS ISDM versus ROI ISDM correlations remained significant after FDR correction. Out of all the Retrospective ELS ISDM versus ROI ISDM correlations, 63.9% were positive, with an average correlation of *r*_*s*_ = 0.019 (*r*_*s*_‐min = −0.107; *r*_*s*_‐max = 0.153) and a median uncorrected *p* value of *p* = 0.33 (*p*‐min = 0.003; *p*‐max = 1). See Supporting Information (Figure S8) for ISDM's for Prospective and Retrospective ELS, *d′*, and the 40 HCP regions with significant IS‐RSA correlations with Prospective ELS.

**FIGURE 4:**
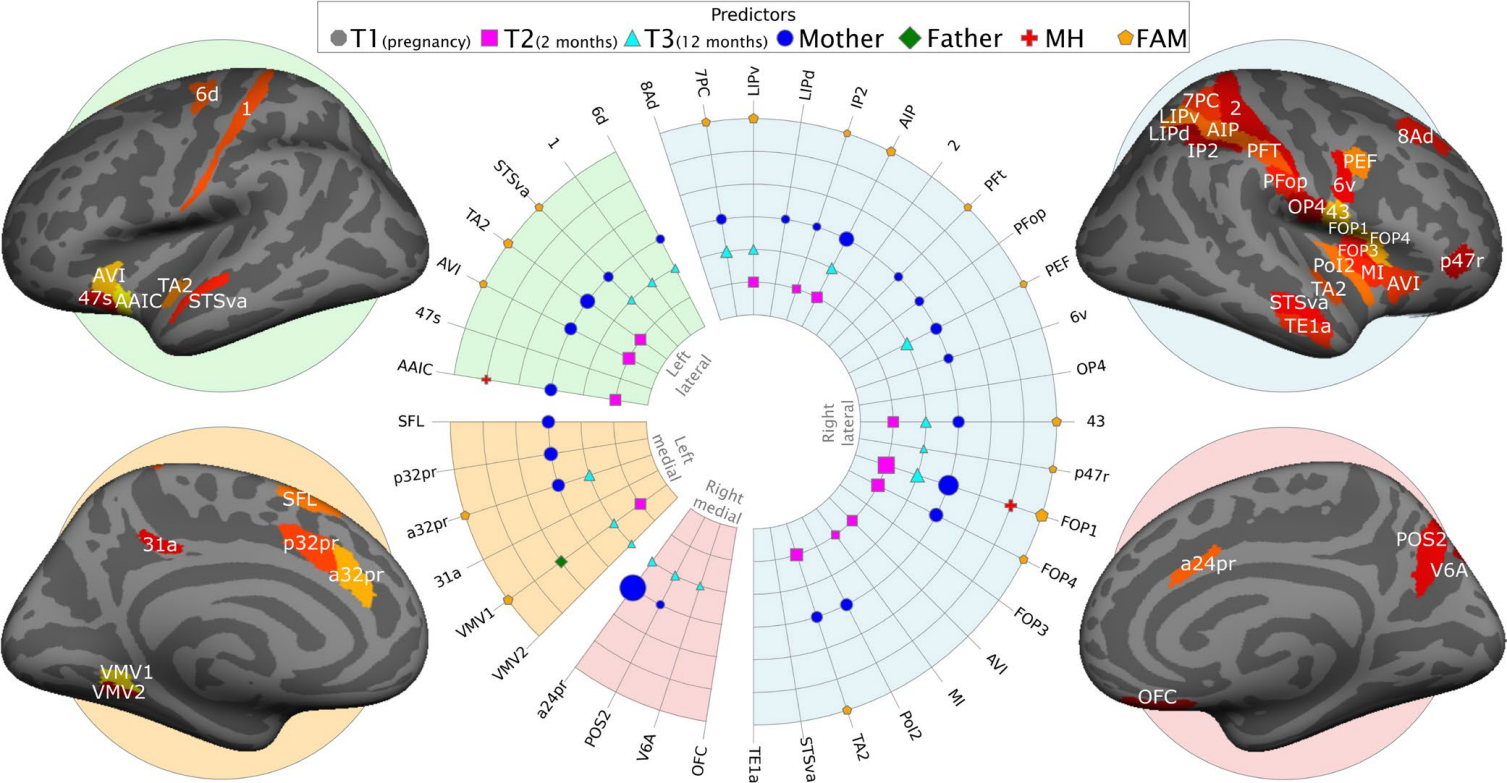
The 40 cortical regions with significant IS‐RSA correlations from the Prospective ELS ISDM versus ROI ISDM partial Spearman's correlation highlighted on inflated medial and lateral surfaces for the right and left hemisphere. The radial plot displays which domains of Prospective ELS had significant IS‐RSA correlations with the 40 regions. In the radial plot, the size of the marker indicates the size of the correlation, with larger markers denoting larger IS‐RSA correlations. The predictors include the domains for timing of ELS (T1: Pregnancy; T2: 2 months; T3: 12 months), reporter (mother or father self‐reports), mental health symptoms (MH), and family relationship problems (FAM).

Next, in order to explore which domains of the Prospective ELS aggregate may have driven the significant associations, each significant ROI specific ISDM from the Prospective ELS ISDM versus ROI ISDM analysis was correlated with ELS ISDMs based on the individual domains of the Prospective ELS aggregate (T1, T2, T3, mother's reports, father's reports, mental health symptoms, family relationship problems). Each correlation between the domain of the aggregate and the RDMs of the significant regions were also tested using a Mantel's test with 5000 permutations, and attained *p* values were FDR corrected to avoid potentially spurious results. None of the ROIs had a significant correlation with ELS reported during the pregnancy (T1) (*r*_*s*_‐min = −0.009; *r*_*s*_‐max = 0.166; *r*_*s*_‐mean = 0.079). Prospective ELS when the child was 2 months old (T2) was significantly associated with 13 regions (*r*_*s*_‐min = 0.081; *r*_*s*_‐max = 0.241; *r*_*s*_‐mean = 0.149), and Prospective ELS when the child was 12 months old (T3) was associated with 16 regions (*r*_*s*_‐min = 0.08; *r*_*s*_‐max = 0.223; *r*_*s*_‐mean = 0.147). Mother's reports were associated with 23 regions (*r*_*s*_‐min = 0.1; *r*_*s*_‐max = 0.288; *r*_*s*_‐mean = 0.173), while father's reports were associated with only 1 region (*r*_*s*_‐min = −0.01; *r*_*s*_‐max = 0.164; *r*_*s*_‐mean = 0.05). Parental mental health symptoms were associated with 2 regions (*r*_*s*_‐min = 0.05; *r*_*s*_‐max = 0.204; *r*_*s*_‐mean = 0.114), while the family relationship problems were associated with 16 regions (*r*_*s*_‐min = 0.061; *r*_*s*_‐max = 0.22; *r*_*s*_‐mean = 0.139). Figure 4 displays the 40 regions with significant Prospective ELS ISDM versus ROI ISDM correlations, in addition to which of the regions had significant correlations with which individual domain of the Prospective ELS aggregate. See Supporting Information (Figure S9) for a depiction of the sorted IS‐RSA correlation values for the correlations between the different ELS domains and the 360 cortical ROI ISDMs.

**FIGURE 5:**
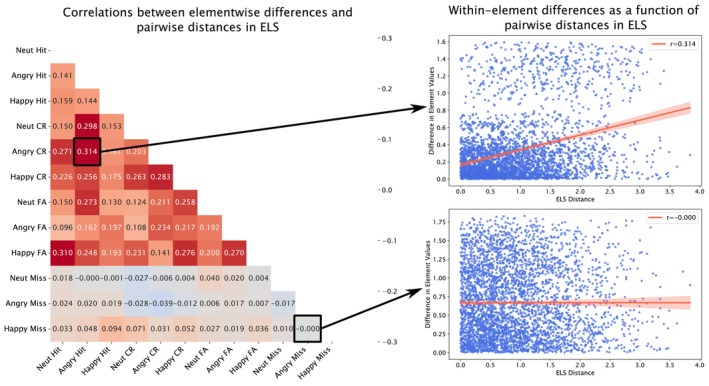
Demonstration for the approach of correlating pairwise dissimilarities in the RDM elements and pairwise dissimilarities in Prospective ELS for the right frontal operculum (FOP1). The matrix (left) displays correlations between each RDM element's absolute differences and ELS dissimilarity, highlighting the direction and consistency of change in pairwise dissimilarities of an element as a function of increasing dissimilarity in Prospective ELS. To attain the matrix, the absolute difference between each unique participant pair's RDM was first calculated, after which each element's absolute difference was Spearman correlated with pairwise differences in Prospective ELS (right). Each datapoint in the scatterplots denotes a unique dyad.

**FIGURE 6:**
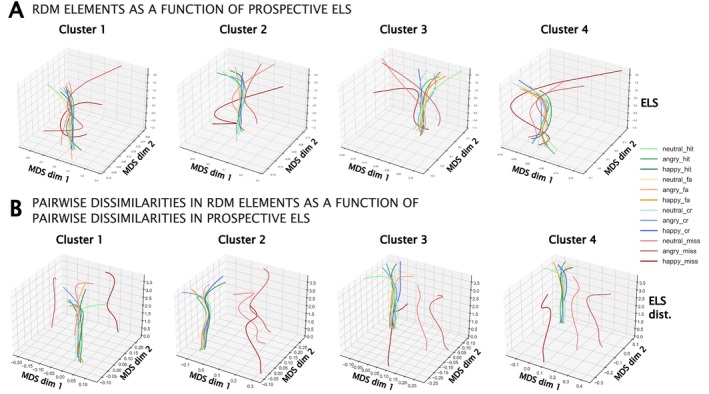
Visualizations for (A) changes in the raw RDM values as a function of Prospective ELS, and (B) changes in difference‐RDMs as a function of pairwise dissimilarities in Prospective ELS. Regions were first combined based on which cluster they belonged to by embedding each RDM (A) or difference‐RDM (B) into two‐dimensional MDS space. Generalized Procrustes Analysis was then used to align the ROIs for (A) each participant or (B) participant pair. These aligned consensus spaces were then sorted according to (A) Prospective ELS (axis “ELS”), or (B) pairwise distances in Prospective ELS (axis “ELS dist.”), after which 3‐ and 5‐degree polynomials were fit through the datapoints corresponding to each unique condition in the RDM (e.g., happy‐hit) respectively.

To better understand the Prospective ELS ISDM versus ROI ISDM IS‐RSA correlations, we utilized several approaches that enabled the visualization of how neural representations changed as a function of Prospective ELS, and how pairwise differences in neural representations changed as a function of pairwise distances in Prospective ELS. First, we calculated Spearman correlations between the pairwise distances in Prospective ELS and pairwise distances in the RDM's elements for each significant region. This approach explored consistency of change in pairwise differences of the RDMs' elements as a function of increasing pairwise dissimilarity in Prospective ELS. Figure 5 shows this approach using the right frontal operculum region (FOP1) that had the strongest IS‐RSA correlation with Prospective ELS. The resulting matrices were then used to construct a ROI‐by‐ROI matrix denoting Procrustes disparities (akin to a distance matrix) by first embedding each of the matrices into a two‐dimensional MDS space and using Procrustes analysis to align the spaces (yielding disparity values for each comparison denoting how far apart the shapes remained after alignment). Ward's method was then employed for hierarchical clustering, yielding four clusters (silhouette score = 0.647). Then, both the raw RDMs and the pairwise difference RDMs of all regions within a cluster were separately embedded into two‐dimensional MDS spaces, and Generalized Procrustes Analysis was used to align them for each participant (or pair of participants for the pairwise difference RDMs). These aligned shapes comprising 12 points denoting each RDM condition (e.g., happy‐hit) were then visualized across the overall Prospective ELS scores and pairwise distances in Prospective ELS separately (using 3‐ and 5‐degree polynomial curves to smooth the data). The resulting visualizations (Figure 6) depict how (1) the neural representations of the emotional go/no‐go task change as a function of Prospective ELS, and (2) how pairwise differences in the neural representations change as a function of pairwise distances in Prospective ELS.

### 3.4 Complementary Similarity Analyses Results

To gauge whether other approaches to defining pairwise distances in Prospective ELS would yield similar or different results (compared to the NN model using the overall Prospective ELS score), four other approaches to calculating pairwise distances were conducted for comparison: (1) item‐wise (20 total items) Prospective ELS Euclidean distances, (2) domain‐wise (7 domains comprising timing, reporting parent, and problem domain) Prospective ELS Euclidean distances, (3) an AnnaK model (pairwise averages of the overall Prospective ELS score), and (4) an interaction between the AnnaK model and the NN model (product between AnnaK and NN). Each model was tested with Mantel's test (10,000 permutations) and FDR corrected separately within each set of 360 tests (i.e., FDR‐corrected per approach, at *α* = 0.05).

**FIGURE 7:**
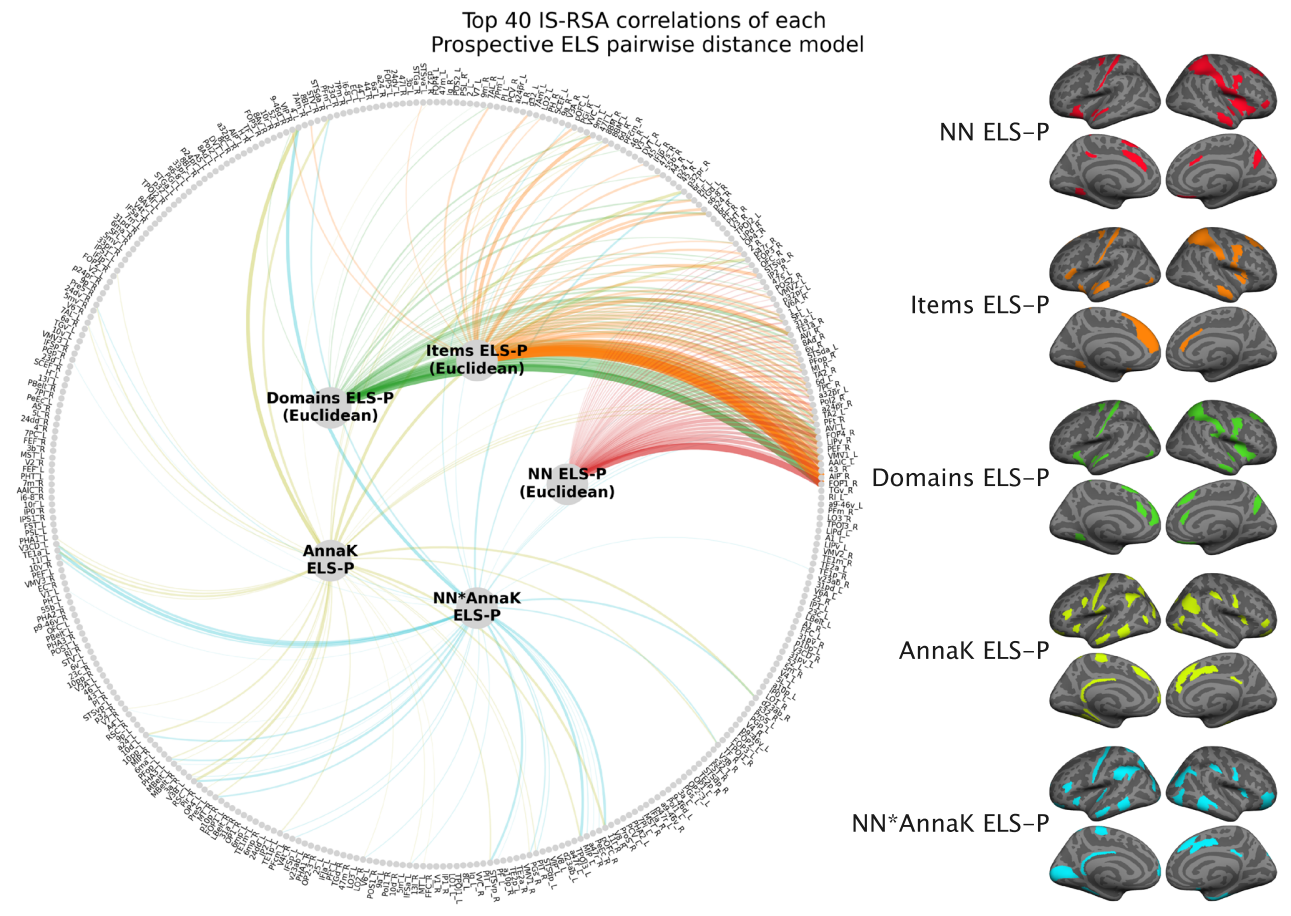
Depiction of the top 40 lowest *p* value IS‐RSA correlations for each approach to defining pairwise distances in Prospective ELS. All IS‐RSA correlations were attained for each pairwise distance approach using the same procedure as in the main interindividual similarity analysis. All 360 parcels from the HCPMMP1.0 on the outer edge of the circle are sorted according to the IS‐RSA correlation *p* values from the main analysis, where the Nearest Neighbor model using the overall Prospective ELS (NN ELS‐P) was used to define pairwise distances. Line thickness indicates the strength of the correlation in relation to all correlations over the pairwise distance approaches. The additional approaches to defining pairwise distances included Items ELS‐P (Euclidean): The Euclidean distance between vectors comprising all 20 Prospective ELS items; Domains ELS‐P (Euclidean): The Euclidean distance between vectors comprising the average Prospective ELS scores of the seven major domains (e.g., all mother's reports, all parent reports when the child was 2 months old, etc.); AnnaK ELS‐P: Pairwise average overall Prospective ELS; NN*AnnaK ELS‐P: The pairwise interaction (product) between overall Prospective ELS Euclidean distance and the pairwise average overall Prospective ELS.

The NN model using the overall Prospective ELS remained the approach with the strongest overall correlations and lowest *p* values across the 360 regions. None of the four complementary approaches to pairwise distances resulted in adjusted *p* values below the alpha level. Nevertheless, the sets of ROIs with the 40 lowest *p* values overlapped substantially with the overall Prospective ELS NN model for the domain‐wise NN model (Dice similarity coefficient, DSC = 0.75; 30/40 ROIs) and the item‐wise NN model (DSC = 0.65; 26/40 ROIs). Overlap was much smaller for the Anna Karenina model (DSC = 0.15; 6/40) and the NN × AnnaK interaction model (DSC = 0.13; 5/40). See Figure 7 for the 40 lowest‐*p* ROIs for each IS‐RSA model.

Finally, due to the null results from the between‐subjects univariate models that included ELS as a predictor, we explored whether IS‐RSA could be used to capture brain function related to between‐subjects effects in clusters derived from the within‐subjects reward model. To this end, we utilized IS‐RSA to inspect whether there was a significant correlation between Prospective or Retrospective ELS ISDM, *d′* ISDM, and the angry FA ISDM. The angry FA ISDM was constructed by first extracting the mean percent signal changes during commission errors to angry facial expressions in seven volumetric clusters (extracted from the within‐subjects reward model), and then calculating pairwise correlation distances between these angry FA profiles for each unique dyad. Just like in the main analysis, partial Spearman's correlation was used, with the same covariates controlled for as in the main analysis. Out of these analyses, only the Prospective ELS ISDM versus *d′* ISDM had a significant IS‐RSA correlation (*r*_*s*_ = 0.101, *p* = 0.016) after FDR correction. However, while the Prospective ELS ISDM versus angry FA ISDM IS‐RSA yielded only a small positive correlation that wasn't statistically significant (*r*_*s*_ = 0.038, *p* = 0.121), we explored whether the pairwise distances in the angry FA ISDM had shared covariance with pairwise distances in the Prospective ELS ISDM and *d′* ISDM. Here, we utilized a combination of IS‐RSA and an associative path model by comparing the observed effect to a distribution of resampled effects. To achieve the resampled effects, both the ELS ISDM and *d′* ISDM were shuffled like in the Mantel's test, and the association was recalculated 5000 times with the shuffled matrices. A significant indirect effect of angry FA on the association between Prospective ELS ISDM and *d′* ISDM (*z*‐direct = 5.029, *p* = 0.065; *z*‐indirect = 5.829, *p* = 0.003; *z*‐total = 6.821, *p* = 0.023) was observed in the analysis.

## 4 Discussion

Uncovering the neurodevelopmental sequelae of early life stress (ELS) remains a challenging yet critical avenue of psychological and neuroscientific research. While numerous studies have documented associations between ELS and altered brain function, findings have yet to converge into clear, generalizable outcomes (Kraaijenvanger et al. 2020). This inconsistency likely reflects the complexity of childhood stress and the underlying neurodevelopmental processes, which may not be adequately captured by traditional univariate analyses that rely on mean activation differences or binary exposure categorizations. Such paradigms often inadvertently overlook the nuanced and gradual effects of chronic ELS.

To expand on previous research, we utilized a novel combination of representational similarity analysis (RSA) and its intersubject variant (IS‐RSA) to investigate whether interindividual similarity in ELS exposure, assessed both prospectively (parental reports of mental health symptoms and family relationship problems during pregnancy and infancy) and retrospectively (participant self‐reported adverse traumatic and stress‐provoking childhood experiences), aligns with interindividual similarity in cortical neural representations during an emotional go/no‐go task in early adulthood. Additionally, we employed typical statistical procedures to investigate behavioral and neural associations, compared the IS‐RSA results to alternative pairwise distance approaches, and explored whether our IS‐RSA results could be further visualized to gain more clarity about the observed effects. This study also sought to examine how different operational definitions of ELS and advanced multivariate approaches contribute to variability in findings, thereby highlighting methodological considerations for future neurodevelopmental research.

The interindividual similarity analyses revealed significant positive correlations between interindividual similarity in Prospective ELS and neural representational similarity in 40 different cortical regions. However, none of the correlations between interindividual similarity in Retrospective ELS and cortical neural representations were significant. Most of the regions with significant correlations between similarities in Prospective ELS and neural representational similarity were observed in the lateral right hemisphere. Notable observed significant regions include the anterior insula, superior temporal sulcus, anterior cingulate cortex, and the right frontal operculum and intraparietal regions.

Many of the regions with significant IS‐RSA correlations in the current study have been implicated in previous research on neurodevelopment, emotions, cognitive control, and facial emotion recognition. For example, the frontal operculum is related to facial emotion control (Caruana et al. 2016), processing (Bayer et al. 2018), and discrimination (Iarrobino et al. 2021). The insula, a laterally obscured cortical region beneath the sylvian fissure that is surrounded by the frontal, temporal, and parietal operculum, has been associated with a variety of different functions, such as interoception, pain processing, vestibular functions, attention, speech, and importantly, emotional experiencing and social cognition (Uddin et al. 2017). Bidirectional insular connectivity extends especially to regions such as the orbitofrontal, anterior cingulate, supplementary motor, primary and secondary somatosensory, and temporal cortices (Gasquoine 2014). Many of these connected regions overlap with several regions with significant IS‐RSA correlations in the current study. There is also brain region‐related agreement between the current study and evidence originating from research spanning over four decades about the importance of the right hemisphere's role in the recognition of emotional facial expressions (Ley and Bryden 1979). A regional overlap can also be seen between the current study and a study by Adolphs et al. (1996) that investigated cortical regions with the largest association with facial emotion recognition impairment in lesion patients. This overlapping of regions suggests both that the IS‐RSA methodology captures functional alterations and that Prospective ELS, thus, likely has a systematic influence on how these specific regions represent facial emotional expressions.

Importantly, that interindividual similarity in Prospective ELS, but not Retrospective ELS, had significant positive correlations with neural representations highlights how moderate but chronic ELS might have a more comprehensive influence on brain development than previously thought. Some previous research has explicitly focused on more moderate forms of ELS (Shapero et al. 2015; Edge et al. 2009), even though a consensus on the proper definition of moderate ELS has yet to emerge. This underscores the necessity of re‐evaluating how moderate ELS is conceptualized and measured in both research and clinical settings. The lack of a clear consensus on its definition may contribute to variability in findings across studies, highlighting the need for standardized criteria that capture the nuanced, cumulative impacts of chronic but moderate ELS. The results of the current study suggest that the assessment of Prospective ELS through parental mental health problems or conflict in marriage may capture age‐salient stressors with detectable neurodevelopmental outcomes.

IS‐RSA correlations between the 40 significant regions and the individual domains of the Prospective ELS index were also inspected. Parental mental health and relationship problems during the child's infancy (when the child was 2 and 12 months old), problems reported by the mother in general (over all timepoints and questionnaire types), and family relationship problems in general (over both parents and all timepoints) had the highest IS‐RSA correlations with the 40 regions. Surprisingly, interindividual similarity in ELS during the pregnancy (2nd trimester) was not strongly associated with interindividual similarity in neural representations in any of the 40 regions, despite the contemporary scientific evidence strongly suggesting the importance of maternal prenatal distress (i.e., prenatal programming) for structural and functional brain development of the child (Wu et al. 2024). The discrepancy in findings may be caused by confounding factors, such as (a) the nature of the emotional go/no‐go task focusing on relatively narrow emotional–cognitive self‐regulation, (b) the fact that few expecting mothers and fathers in the current study reported extremely high levels of distress symptoms, or (c) the inclusion of the father's responses in the aggregate. Nevertheless, the results of the current study suggest that ELS relating especially to maternal and family‐related problems during the first year of life might play a large role in shaping cortical representations of facial emotion processing, emotion regulation, and cognitive control.

Because emotion regulation problems and impulsive behavior have been implicated in those with experiences of ELS (Sanchez and Bangasser 2022; McMullin et al. 2021), we investigate whether seven clusters displaying significantly different activity during presentations of angry facial expressions (extracted from a within‐subjects model) would also display similar activation during commission errors to angry faces in those with similar ELS exposure. However, similarity in neither Prospective nor Retrospective ELS was significantly associated with similarity in the activation of these regions. Nevertheless, because a significant association between similarities in Prospective ELS and detection sensitivity (*d′*) was observed, we additionally explored whether similarity in the activation profiles mediated this association. We found that similarity in the activation profiles did mediate the relationship between similarity in Prospective ELS and similarity in detection sensitivity. This finding suggests shared variance in interindividual similarities in Prospective ELS, brain activity, and behavioral responses, highlighting again the potential long‐term influences that even more moderate but chronic forms of ELS may carry into early adulthood.

The most straightforward interpretation of these IS‐RSA results is that individuals who are more similar in terms of Prospective ELS are also more similar in terms of how their brain represents socio‐emotional information and related self‐regulation. The results thus suggest that experiences during the first year of life related to parental mental health and family relationship problems may influence the development of a wide range of cortical regions in how they process socio‐emotional information and coordinate relevant cognitive‐emotional self‐regulation. However, due to the primary focus on the multidimensional nature of the approaches utilized in the present study, the aspects of the representational structure underlying the observed effects remain relatively obscured.

Nevertheless, our attempts to visualize the associations between interindividual similarities in Prospective ELS and the RDMs provided unique findings. First, there was a surprisingly strong clustering of the regions with the strongest IS‐RSA correlations (see Figure S5). Importantly, this clustering emerged due to two congruent approaches: (1) the transformation of the representational dissimilarity matrices into matrices that denote correlations between each RDM element's pairwise differences and pairwise differences in Prospective ELS, and (2) the utilization of multidimensional scaling and Generalized Procrustes Analysis disparities for inspecting between‐region dissimilarities. While much work remains to be done to untangle the reason as to why this approach yielded such strong clustering, it suggests that such an approach might be useful for studying how neural representations are shaped by any suspected predictors. By clustering regions based on Procrustes disparities using matrices denoting correlations between pairwise differences in the RDM cells and pairwise differences in the predictor, one is essentially clustering based on how a region's neural representations are shifted as a function of the predictor, while respecting the multidimensional nature of neural representations. Based on the strong clustering that emerged in the present study, ELS seems to contribute to rather specific changes in neural representations of actionable social–emotional information processing in specific but diffuse regions, instead of an influence that changes in a graded manner based on anatomical proximity. While precise disentangling of the underlying reason for the observed clustering is beyond the scope of the current study, we suspect that it may reflect how ELS alters expectations, perceptions, goal orientation, and response strategy in social–emotional contexts. ELS likely shifts how the brain represents social–emotional information to differentiate certain emotions more easily and prefers certain response types (omission vs. commission). ELS might sensitize individuals to, for example, expect and more easily recognize specific emotions (such as anger) and to more readily commit to perceiving them in others.

The visualizations created using Procrustes‐aligned multidimensional scaling on the raw values of the RDMs (Figure 6A) demonstrate that there are some clear patterns in the neural representational spaces as a function of Prospective ELS. First, almost all the different conditions in the representational space seem to converge and develop similarly as a function of ELS. Additionally, those with low ELS seem to have a slightly more varied neural representational structure. The exception to this pattern is another pattern, which is that omission errors are consistently dissimilar to most other conditions across the different regions, and they do not display consistent patterns as a function of ELS across different clusters of regions. When looking at how interindividual dissimilarities in the RDMs change as a function of interindividual dissimilarity in Prospective ELS (Figure 6B), a common pattern emerges: dissimilarities in neural representations as a function of dissimilarities in ELS remain relatively stable but disperse when pairwise differences in ELS become large. This suggests that there is noticeable consistency in how ELS relates to changes in neural representations overall, and that this consistency breaks only at extreme pairwise dissimilarities in ELS.

In our complementary analyses, we explored whether different approaches to defining interindividual similarities would yield similar results to our primary Nearest Neighbor model where only absolute arithmetic differences in the overall Prospective ELS scores were used. Surprisingly, IS‐RSA correlations were notably smaller and non‐significant across the 360 regions when constructing interindividual similarities based on either the major domains or the individual items comprising Prospective ELS. Absolute pairwise differences in the overall Prospective ELS score thus remained the most reliable approach to defining Euclidean distances for Prospective ELS, perhaps due to reduced noisiness of the overall score compared to the domains or the individual items. There was, however, noticeable agreement with which regions these different approaches to interindividual similarities had the strongest correlations, suggesting they are ultimately telling a similar story (Figure 7).

We additionally explored whether an Anna Karenina model, or an interaction between the Nearest Neighbor and Anna Karenina model, would yield different IS‐RSA results. Importantly, Anna Karenina, in contrast to the Nearest Neighbor model, retains information about the absolute level of ELS, as it denotes the average ELS of a pair. Despite previous literature suggesting the benefit of the Anna Karenina model (Finn et al. 2020), no significant correlations were attained using either the Anna Karenina model or the interaction between Anna Karenina and Nearest Neighbor. However, as with the different approaches to defining the Nearest Neighbor pairwise distances, the regions with high correlations using an Anna Karenina model largely agree with the regions with high correlations in the Nearest Neighbor models. The weaker correlations using Anna Karenina models suggest that meaningful observable differences in neural representations as a function of Prospective ELS emerge more through absolute pairwise differences irrespective of the actual level of ELS. A pair that has low ELS and a pair that has high ELS both demonstrate similar differences in their neural representations as long as the individuals in these pairs are similarly far apart from each other within their respective dyads. This likely does not mean that there are no differences in neural representations between those with low ELS and those with high ELS, but that the meaningful differences in neural representations might exist in differences within the measure. Thus, quanta attained through operationalizations of complex psychological constructs like ELS might not always translate to hard objective outcomes in the brain, but meaningful associations to brain function (and perhaps structure) may still emerge within differences between individuals in the operationalized construct.

Unlike the similarity analyses, the univariate whole‐brain analyses did not yield significant ELS‐related between‐subjects differences. However, clear main effects for response correctness, motor response, valence of the stimulus, and the two‐way interactions of correctness and valence with motor response were observed within‐subjects. The difference in results between the whole‐brain repeated measures approach and the IS‐RSA approach suggests that univariate analyses might not be as sensitive in capturing complex differences in brain function.

As for the behavioral data, an interaction effect emerged between Prospective ELS and the mother's age, which suggested that the deleterious influence of Prospective ELS on detection sensitivity was not as large in those with older mothers. This interaction suggests that the potentially decremental effect of ELS on the ability to accurately recognize facial expressions and interpret social–emotional information might be attenuated in those with older mothers. The simplest interpretation of this result is that older mothers, compared to younger mothers, may have life experiences that benefit the developing child when it comes to coping with the parent‐related mental health and family‐related problems. As briefly mentioned earlier, a significant positive correlation was also observed between interindividual similarity in Prospective ELS and interindividual similarity in detection sensitivity using IS‐RSA. No association, however, between ACEs and detection sensitivity was observed in any of the models employed. Also, no differences in reaction time or criterion due to Prospective or Retrospective ELS were observed, suggesting that the cognitive faculties coordinating these behavioral aspects are not as strongly influenced by ELS as those coordinating detection sensitivity.

One of the narratives underlying the developmental alterations following ELS is that exposure to prolonged or extreme stress during early childhood results in biological cascades stemming from overt activation of the stress response system, disrupting the individual's homeodynamic balance (Agorastos et al. 2019). Despite the generally grim narratives underlying the deleterious outcomes of ELS, these outcomes can also be viewed as expectable adaptations that help the individual survive in their early environment, shaping rather than overtly impairing cognition (Frankenhuis and de Weerth 2013). In line with this, the use of neural representations (as opposed to mere brain activation) as the outcome, the IS‐RSA methodology, and the post‐results inspections utilized in the present study do not confer inferences about performance or health‐related normativity. Instead, they merely allow the inspection of within‐sample similarity structures free from judgments about what should or should not be. That interindividual similarity in Prospective ELS was associated with interindividual similarity in neural representations does not inform us about whether a specific representational structure is inherently desirable. Instead, the result highlights a somewhat continuous link between prospectively measured moderate–chronic ELS and brain function in an analytically robust way, due to being less constrained by assumptions about the underlying nature of brain function. This approach is contrasted to frameworks such as the univariate activation analysis framework, with which no significant between‐subjects differences could be uncovered in the current study. Thus, the current study demonstrates how a combination of RSA and IS‐RSA, when paired with spatial and shape analytical approaches like multidimensional scaling and Procrustes analysis, offers legitimate aid in untangling the complex brain function‐related outcomes of ELS. Importantly, these approaches are not limited to developmental neuroscience and ELS research but can be extended to almost any variables of interest.

The findings of this study have broader implications for understanding the systemic influences of ELS on brain development without assigning normative judgments to specific neural or behavioral outcomes. The IS‐RSA association between Prospective ELS and neural representational similarity demonstrates how chronic, moderate ELS exposure relating to parental mental health and family relationship problems during pregnancy and infancy shapes brain function in a systemic and robust, but multidimensional, manner. The observed systematic interindividual differences in the neural representational structures may reflect adaptive responses to early environments and could therefore serve protective functions, in line with theory surrounding the formation of early attachment. This perspective leans on the notions of resilience and plasticity of the human brain, emphasizing its ability to organize itself in response to chronic stress‐inducing environments that pose unique demands for the child. The results also underscore the importance of considering moderate but chronic ELS domains, such as parental mental health and family relationship problems, in clinical settings. While extreme but transient ELS events remain central developmental disruptors, the results of the current study highlight the potential long‐term developmental influence of chronic but moderate ELS, while implicitly shedding light on how human children develop over time into idiosyncratic individuals.

Nevertheless, the need for more precise research undoubtedly remains to uncover how ELS influences brain development. For example, in the current study, family relationship problems were not evaluated during pregnancy due to the Prospective ELS questionnaires including questions about the parent–child relationship. While the sample was balanced for Prospective ELS due to the stratified sampling, few individuals displayed high ACEs in the Retrospective ELS measurement. This lack of high Retrospective ELS participants might at least partly explain why no significant results were obtained for it. Alternatively, the ACEs questionnaire concerns adverse events that have occurred also during middle and late childhood. The lack of systematic effects relating to Retrospective ELS in the current study might thus be due to (a) large within‐sample variation in the timing of adverse events over the whole childhood, (b) a lack of participants with adverse events during the early sensitive periods, or (c) a lack of variability in ACEs between participants. Future research would benefit from varied and rigorous operationalizations of ELS, perhaps by examining multiple aspects of distress (e.g., neglect vs. threat; McLaughlin and Sheridan 2016) over various timepoints. Finally, as with many tasks utilized in psychological experiments, the emotional go/no‐go task captures complex processes, some of which are likely yet to even be conceptualized. The structure employed for the representational dissimilarity matrices in the current study included both the quality of the emotional stimuli and the signal‐detection theory‐derived behavioral outcomes and is thus a complex outcome measure irreducible to singular conceptualizations like emotion regulation or cognitive control. Future research would benefit from the disambiguation of neural representational structures (and ELS‐related changes within them) by utilizing simpler tasks that unanimously capture the underlying psychological construct of interest.

In conclusion, we utilized a combination of RSA and IS‐RSA alongside spatial and shape analytical approaches to demonstrate complex associations between prospectively measured moderate ELS and relatively widespread cortical neural representations during an emotional go/no‐go task in early adulthood. Spatial and shape analytical approaches revealed systematic patterns in neural representational spaces as a function of ELS, demonstrating their potential utility for future neuroscientific and developmental research. The unique sample of Finnish families followed since pregnancy enabled a bridging between the prenatal period and early adulthood of the participants, elucidating the far‐reaching implications ELS may have on adult brain function. The utilization of different measurements of ELS informs future research about the use of various operationalizations and how they might influence observable outcomes. The difference in results between univariate activation analyses and IS‐RSA demonstrated in the current study also highlights the need for researchers to adopt multivariate approaches that take into consideration the complexities of brain function.

## Author Contributions

M.I.: Conceptualization, methodology, software, validation, formal analysis, data curation, writing – original draft, writing – review and editing, visualization, funding acquisition. J.L.: Conceptualization, investigation, data curation, writing – review and editing, supervision, project administration, funding acquisition. M.F.: investigation, data curation, writing – review and editing. M.V.: investigation, data curation, writing – review and editing. R.‐L.P.: conceptualization, investigation, resources, writing – review and editing, supervision, project administration, funding acquisition. P.W.: conceptualization, methodology, software, validation, investigation, data curation, writing – review and editing, supervision, project administration.

## Ethics Statement

The studies involving human participants were reviewed and approved by the Ethics Committee of the Hospital District of Helsinki and Uusimaa, Finland, and conducted in accordance with the Declaration of Helsinki. Written informed consent to participate in this study was provided by the participants, their legal guardian, or next of kin, and they were monetarily compensated for their time (15 €/h).

## Conflicts of Interest

The authors declare no conflicts of interest.

## Supporting information


**FIGURE S1:** Histograms depicting frequencies of scores for each individual item comprising the overall Prospective ELS scores. Items cover timing (T1: pregnancy (2nd trimester); T2: child at 2 months old; T3: child at 12 months old), parent (mother's reports; father's reports), and questionnaire (GHQ: General Health Questionnaire; BDI: Beck's Depression Inventory; PSI: Parenting Stress Index; DAS: Dyadic Adjustment Scale).

**FIGURE S2:** Scatterplots with fitted lines depicting associations between the overall Prospective ELS score and the individual items comprising the overall Prospective ELS score.

**FIGURE S3:** Scatterplot with fitted line depicting the association between overall Prospective ELS and Retrospective ELS scores.

**FIGURE S4:** Visualizations for the averages (mean) and standard deviations of each RDM element depicting Spearman's correlations between pairwise dissimilarities in the RDM element and pairwise dissimilarity in Prospective ELS. To attain the matrices, first, the RDMs' absolute element‐wise differences were calculated for each unique pair of participants within each ROI that had a significant IS‐RSA correlation (yielding difference‐RDMs). Then, within each ROI, a Spearman correlation was calculated between the difference‐RDM elements and pairwise dissimilarity in Prospective ELS, yielding a matrix whose elements denote the direction and consistency of change in that element's pairwise dissimilarities as a function of Prospective ELS dissimilarity. Finally, the mean and standard deviation values of each element of these matrices were calculated. The resulting matrices thus denote which elements of the difference‐RDM change most strongly and consistently as a function of pairwise dissimilarities in Prospective ELS, and how much variation there is in this strength and consistency within each of the four clusters.

**FIGURE S5:** Visualization for the clustered (Ward's method) ROI‐by‐ROI Procrustes disparity matrix with dendrograms, and surface projection of the regions with cluster‐based color coding, for the 40 regions with significant IS‐RSA correlations between pairwise distances in Prospective ELS and the RDMs. Clustering was achieved by computing for each significant region a matrix whose cells denote correlations between pairwise distances in the region's RDM elements and pairwise distances in Prospective ELS. These matrices were then turned into correlation distances (1−*r* for each cell) for MDS embedding, and Procrustes analysis was employed. Procrustes analysis finds the optimal transformation that minimizes the sums of squares of the point‐wise differences between the MDS shapes, and the residuals are then used as an indicator of disparity between the shapes (akin to a distance metric).

**FIGURE S6:** Schematic for the ELS ISDM versus Angry FA ISDM analysis steps. (A) Shows the seven extracted volumetric regions (color coded) from the univariate within‐subjects reward model. (B) Displays example activation profiles of the average signal changes (compared to the mean functional signal) during commission errors to angry facial expression presentations that were extracted for each cluster for each participant. (C) Pairwise distances between vectors of the profile values were then calculated using correlation distance and mapped onto an intersubject dissimilarity matrix, which was correlated with ELS ISDM and *d*′ ISDM with Spearman's correlation, both separately and in an associative path model, while controlling for mother's age and SES, participant sex, and ART‐status (each with their own ISDM).

**FIGURE S7:** Cortical surface projections of the within‐subjects main effects (1000 permutations, *p* = 0.005 cluster *p* threshold; *p* = 0.001 cluster forming *p* threshold) on inflated left and right hemisphere surfaces. Sulci are highlighted in dark grey, gyri in light grey. Displayed are main effects of correctness (correct vs. incorrect trials; Fmin = 40, Fmax = 100), motor response (response vs. nonresponse trials; Fmin = 15, Fmax = 50), valence (happy vs. angry vs. neutral facial expression stimuli trials; Fmin = 9, Fmax = 20), and interactions for correctness × motor response (Fmin = 9, Fmax = 70) and valence × motor response (Fmin = 9; Fmax = 20).

**FIGURE S8:** Visualizations of ISDMs for Prospective ELS (overall score), Retrospective ELS, *d*‐prime, and the 40 regions with significant IS‐RSA correlations between pairwise distances in Prospective ELS and pairwise (correlation) distance in RDMs. ISDMs are sorted according to Prospective ELS.

**FIGURE S9:** Sorted correlation coefficients for intersubject representational similarity analysis correlations between different ELS measurements and the 360 ROI specific RDMs. Correlations have been sorted within each ELS measurement to demonstrate the distribution and ratio of negative and positive correlations. Correlations were attained from partial Spearman's correlation between the vectorized intersubject dissimilarity matrices constructed from various ELS measurements (Prospective and Retrospective ELS, and the 7 different domains of the Prospective ELS), and the 360 intersubject dissimilarity matrices constructed from pairwise correlation distances between vectorized representational dissimilarity matrices for each cortical region of the Human Connectome Project Multimodal Parcellation 1.0. Correlations depicted include: Prospective ELS (overall Prospective ELS score), Retrospective ELS (adapted revised Adverse Childhood Experiences questionnaire), Pregnancy (Prospective ELS parent reports in pregnancy over all questionnaires), 2 months (Prospective ELS parent reports when the child was 2 months old over all questionnaires), 12 months (Prospective ELS parent reports when the child was 12 months old over all questionnaires), Mother (mother's Prospective ELS reports over all timepoints and questionnaires), Father (father's prospective ELS reports over all timepoints and questionnaires), Mental health (BDI and GHQ from both parents over all timepoints), Family related issues (DAS and PSI from both parents over all timepoints).

## Data Availability

Within‐subjects fMRI result images, cluster masks, and analysis codes are available on the Open Science Framework (https://osf.io/j6bh2/). The raw data from the ongoing longitudinal study project is not readily available because participant privacy and ethical permissions do not allow public sharing of the data. Requests to access the datasets should be directed to R.‐L.P., raija-leena.punamaki-gitai@tuni.fi.
