# Supplementary figures and images for: Similarity in Early Life Stress Exposure Is Associated With Similarity in Neural Representations in Early Adulthood
Source: Hum Brain Mapp. 2025 Oct 4;46(14):e70373. doi: 10.1002/hbm.70373 (PMC12495272; doi:10.1002/hbm.70373)

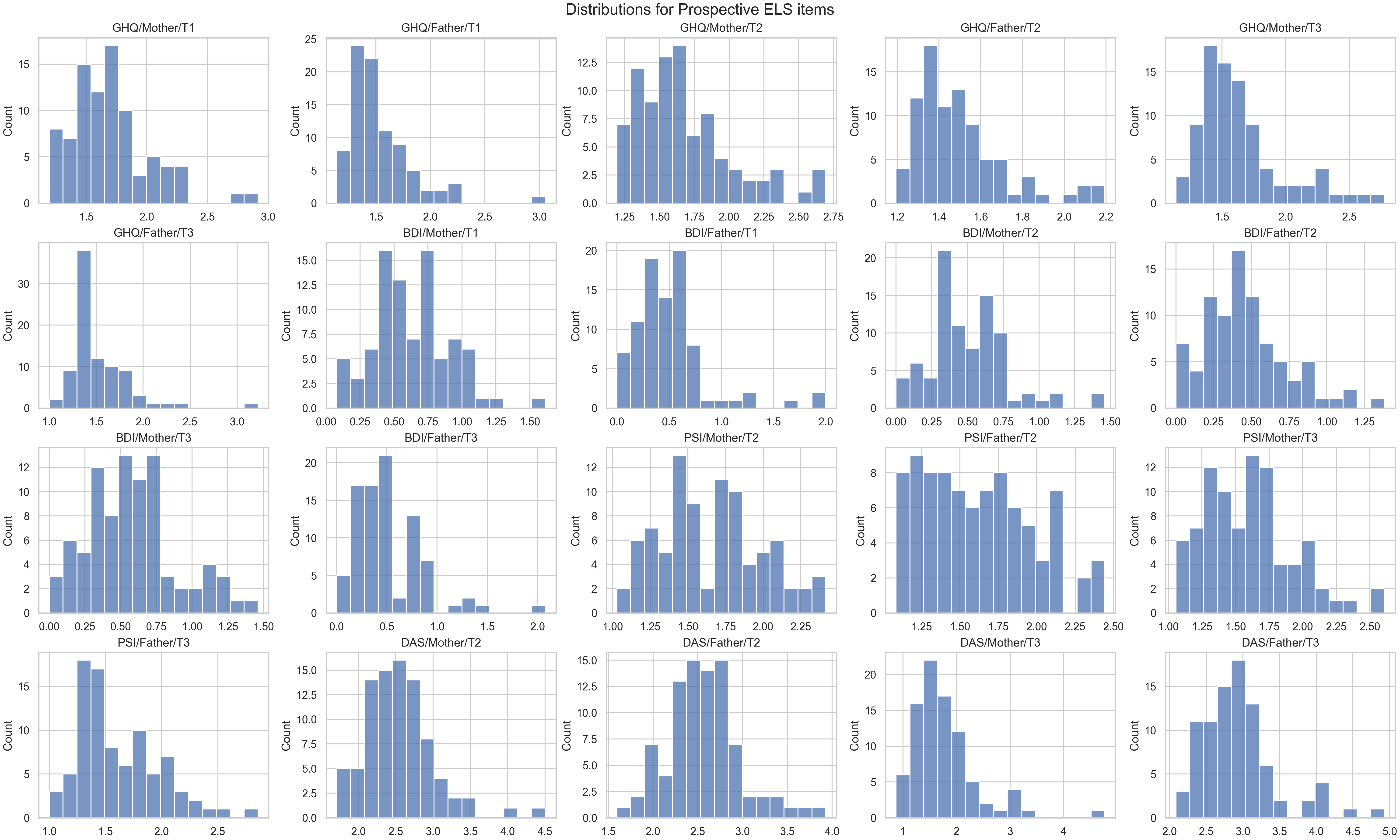

Supplement: Supplementary file 1 — FIGURE S1: Histograms depicting frequencies of scores for each individual item comprising the overall Prospective ELS scores. Items cover timing (T1: pregnancy (2nd trimester); T2: child at 2 months old; T3: child at 12 months old), parent (mother's reports; father's reports), and questionnaire (GHQ: General Health Questionnaire; BDI: Beck's Depression Inventory; PSI: Parenting Stress Index; DAS: Dyadic Adjustment Scale). [file HBM-46-e70373-s009.png]

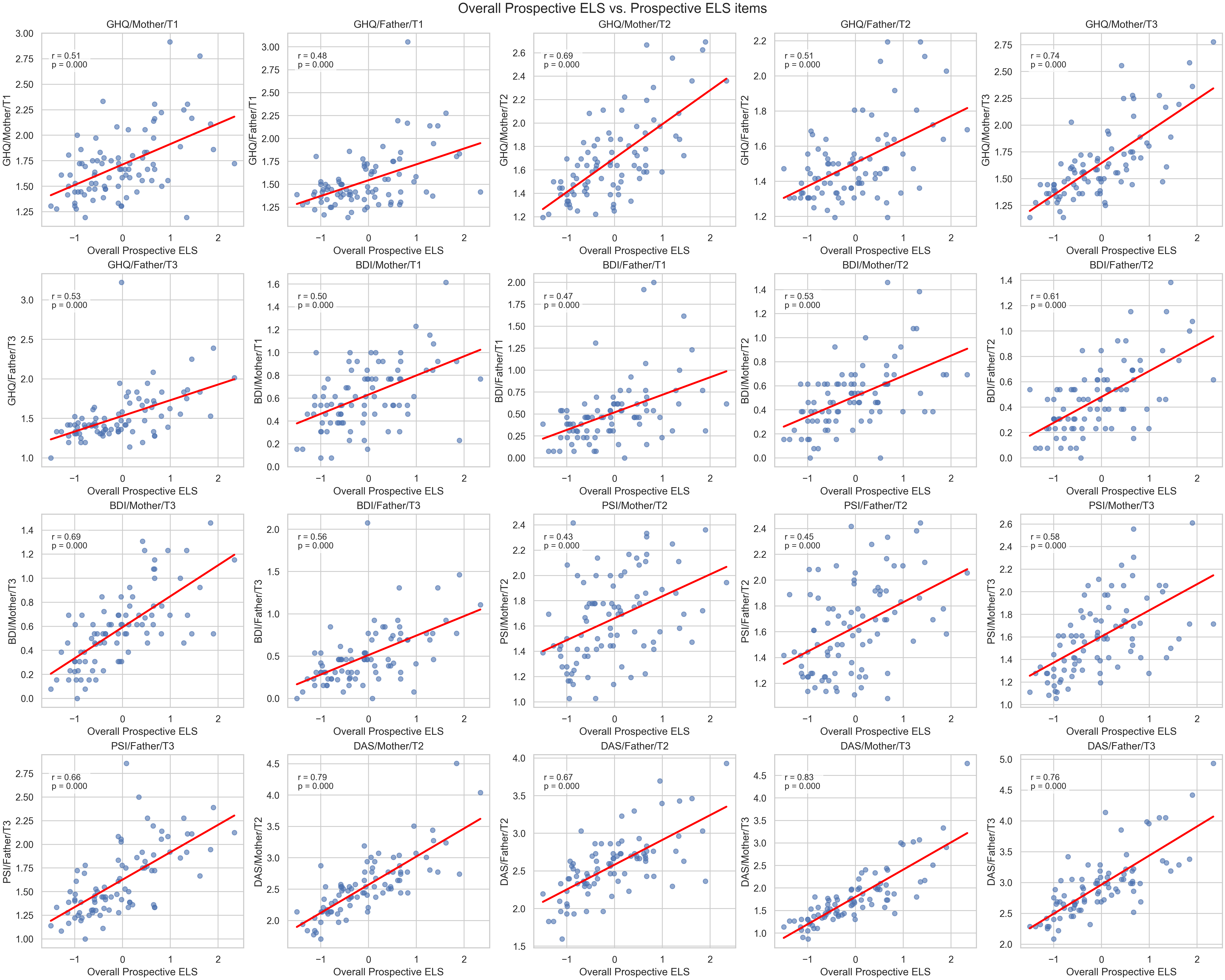

Supplement: Supplementary file 2 — FIGURE S2: Scatterplots with fitted lines depicting associations between the overall Prospective ELS score and the individual items comprising the overall Prospective ELS score. [file HBM-46-e70373-s005.png]

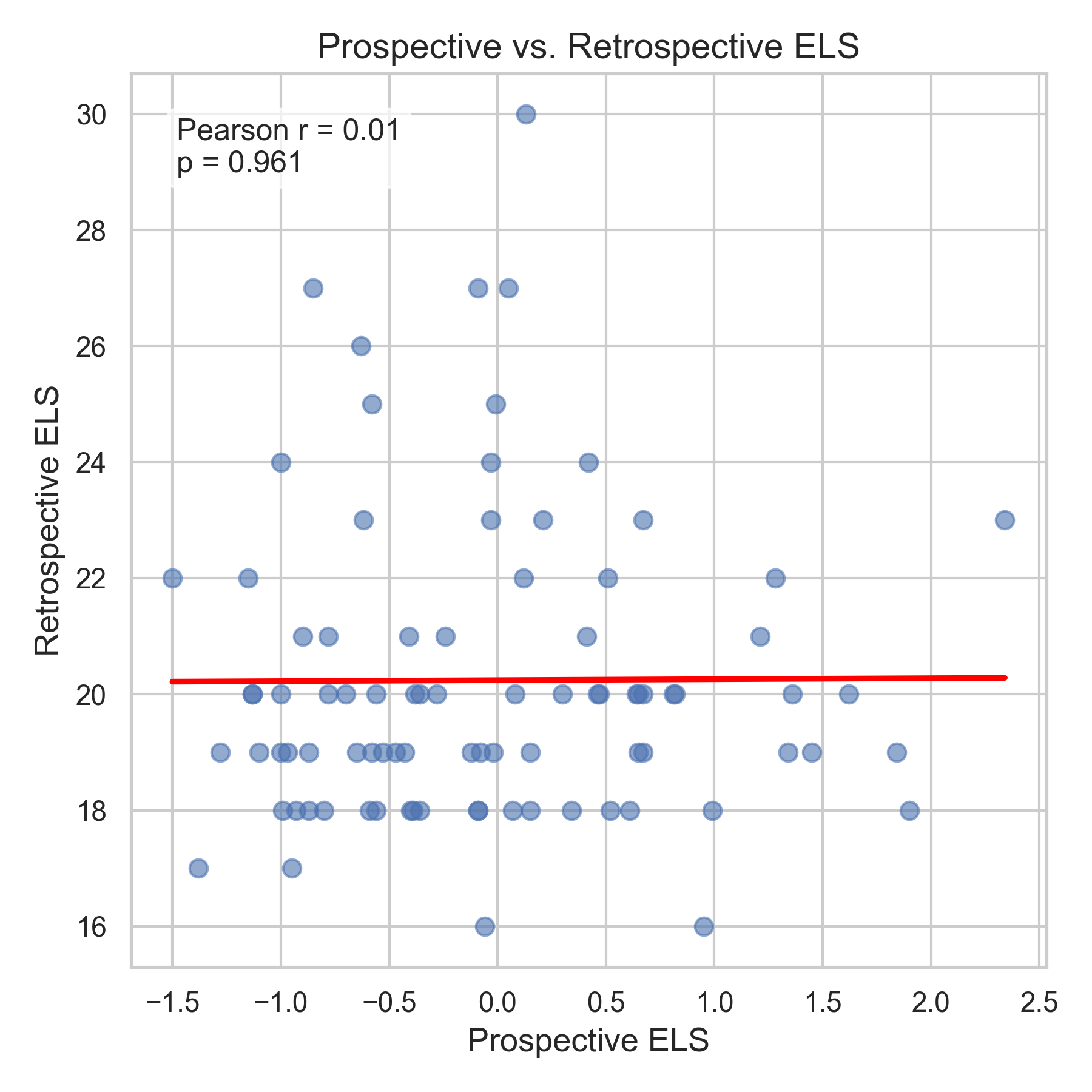

Supplement: Supplementary file 3 — FIGURE S3: Scatterplot with fitted line depicting the association between overall Prospective ELS and Retrospective ELS scores. [file HBM-46-e70373-s002.png]

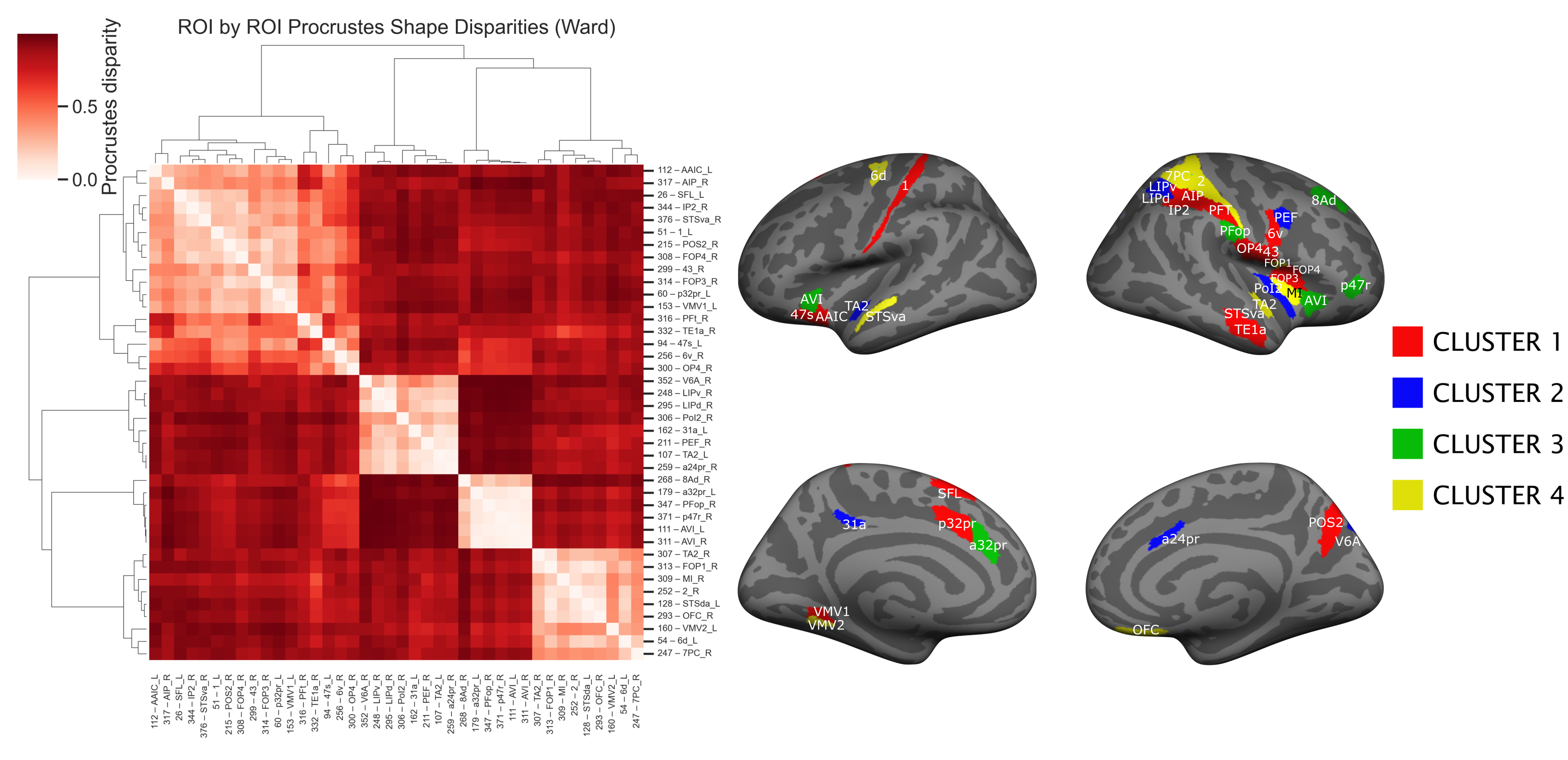

Supplement: Supplementary file 5 — FIGURE S5: Visualization for the clustered (Ward's method) ROI‐by‐ROI Procrustes disparity matrix with dendrograms, and surface projection of the regions with cluster‐based color coding, for the 40 regions with significant IS‐RSA correlations between pairwise distances in Prospective ELS and the RDMs. Clustering was achieved by computing for each significant region a matrix whose cells denote correlations between pairwise distances in the region's RDM elements and pairwise distances in Prospective ELS. These matrices were then turned into correlation distances (1−r for each cell) for MDS embedding, and Procrustes analysis was employed. Procrustes analysis finds the optimal transformation that minimizes the sums of squares of the point‐wise differences between the MDS shapes, and the residuals are then used as an indicator of disparity between the shapes (akin to a distance metric). [file HBM-46-e70373-s006.png]

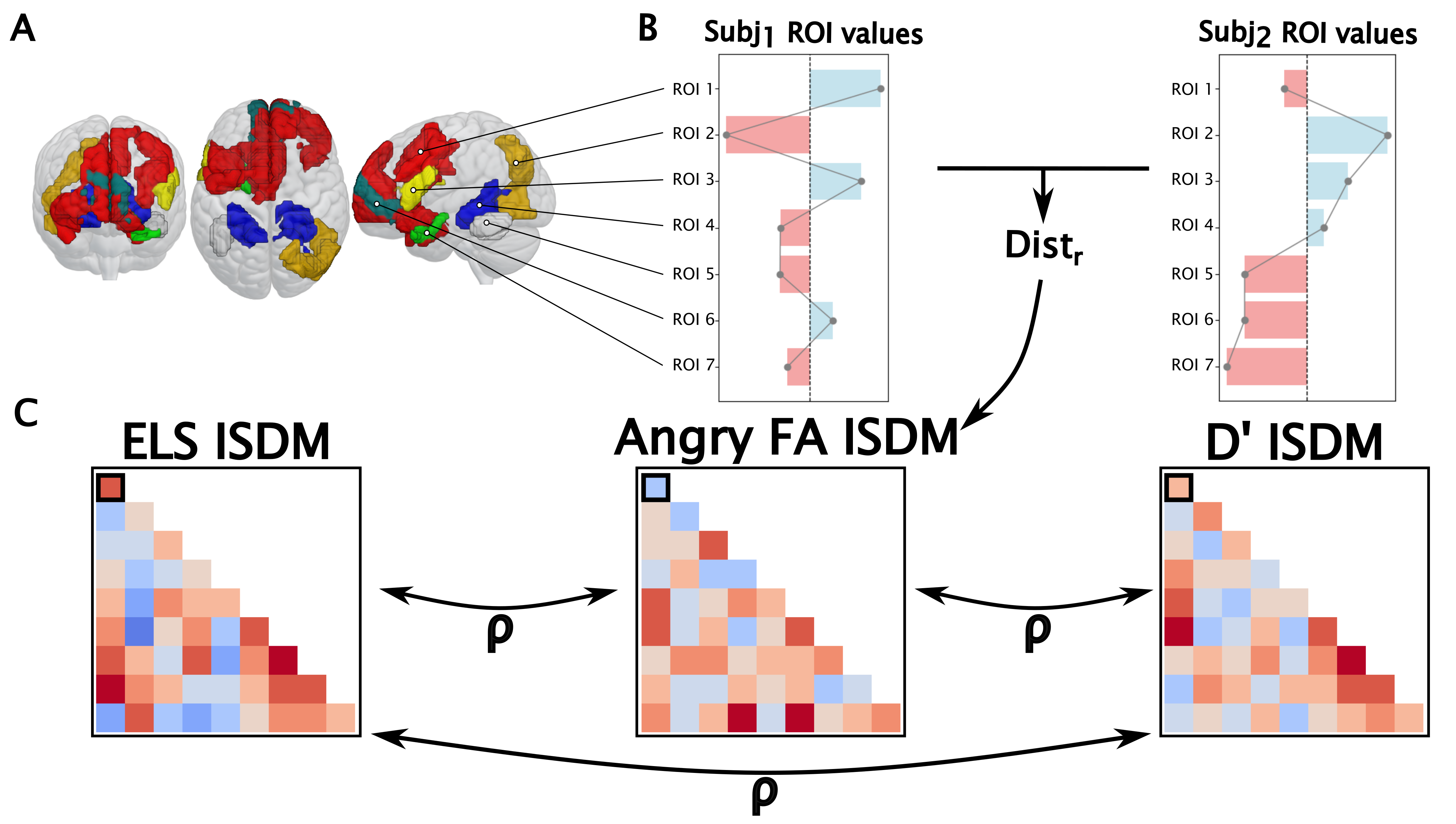

Supplement: Supplementary file 6 — FIGURE S6: Schematic for the ELS ISDM versus Angry FA ISDM analysis steps. (A) Shows the seven extracted volumetric regions (color coded) from the univariate within‐subjects reward model. (B) Displays example activation profiles of the average signal changes (compared to the mean functional signal) during commission errors to angry facial expression presentations that were extracted for each cluster for each participant. (C) Pairwise distances between vectors of the profile values were then calculated using correlation distance and mapped onto an intersubject dissimilarity matrix, which was correlated with ELS ISDM and d′ ISDM with Spearman's correlation, both separately and in an associative path model, while controlling for mother's age and SES, participant sex, and ART‐status (each with their own ISDM). [file HBM-46-e70373-s004.png]

Correctness

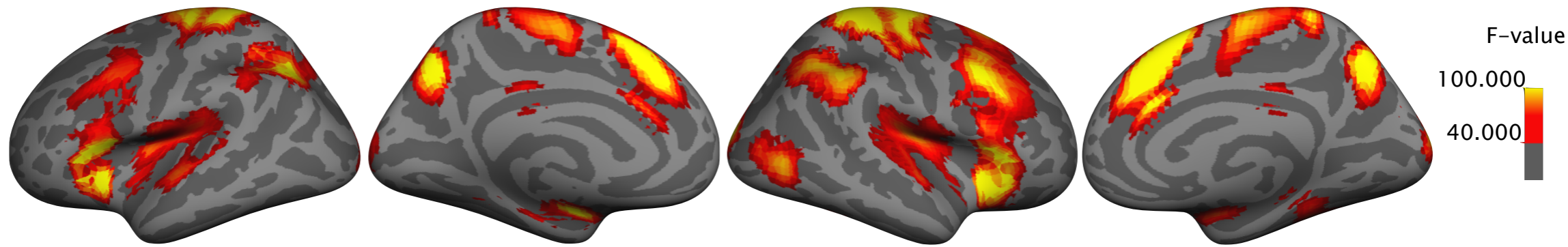

Motor  
Response

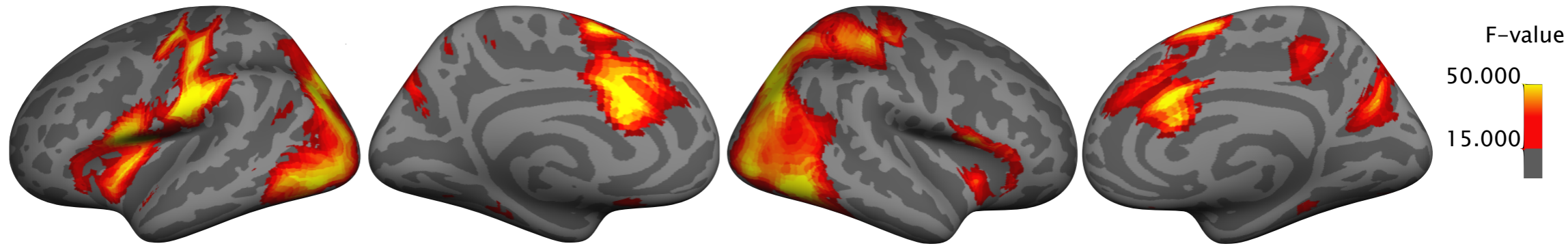

Valence

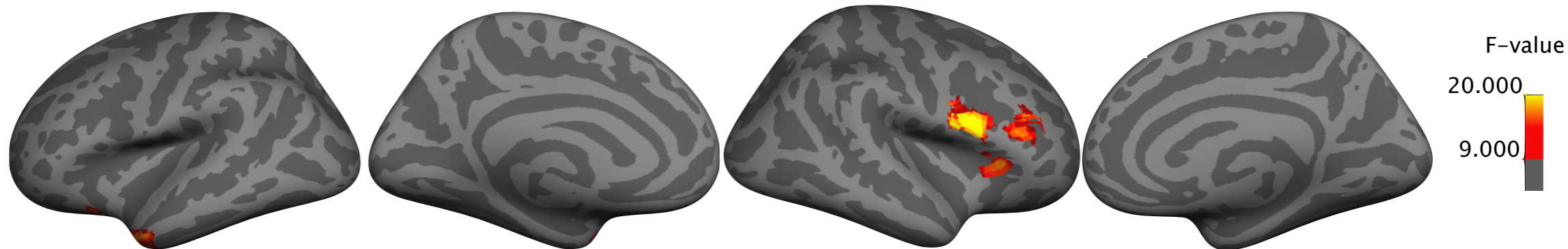

Correctness  
x  
Motor  
Response

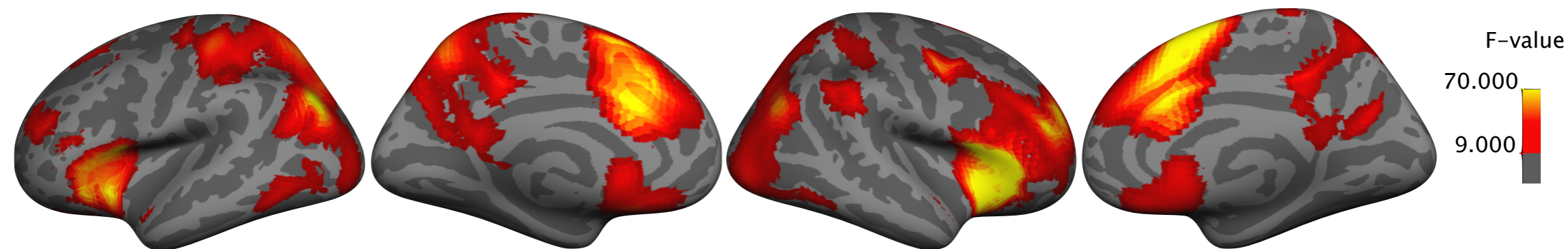

Valence  
x  
Motor  
Response

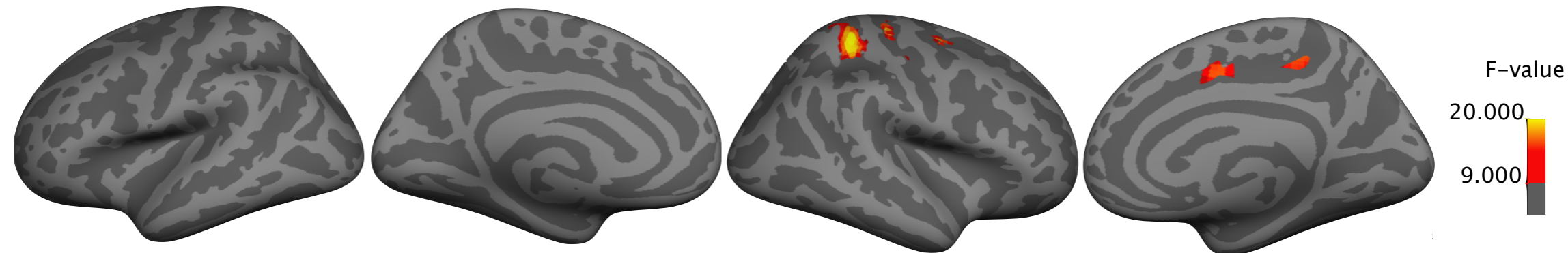

Supplement: Supplementary file 7 — FIGURE S7: Cortical surface projections of the within‐subjects main effects (1000 permutations, p = 0.005 cluster p threshold; p = 0.001 cluster forming p threshold) on inflated left and right hemisphere surfaces. Sulci are highlighted in dark grey, gyri in light grey. Displayed are main effects of correctness (correct vs. incorrect trials; Fmin = 40, Fmax = 100), motor response (response vs. nonresponse trials; Fmin = 15, Fmax = 50), valence (happy vs. angry vs. neutral facial expression stimuli trials; Fmin = 9, Fmax = 20), and interactions for correctness × motor response (Fmin = 9, Fmax = 70) and valence × motor response (Fmin = 9; Fmax = 20). [file HBM-46-e70373-s008.pdf]

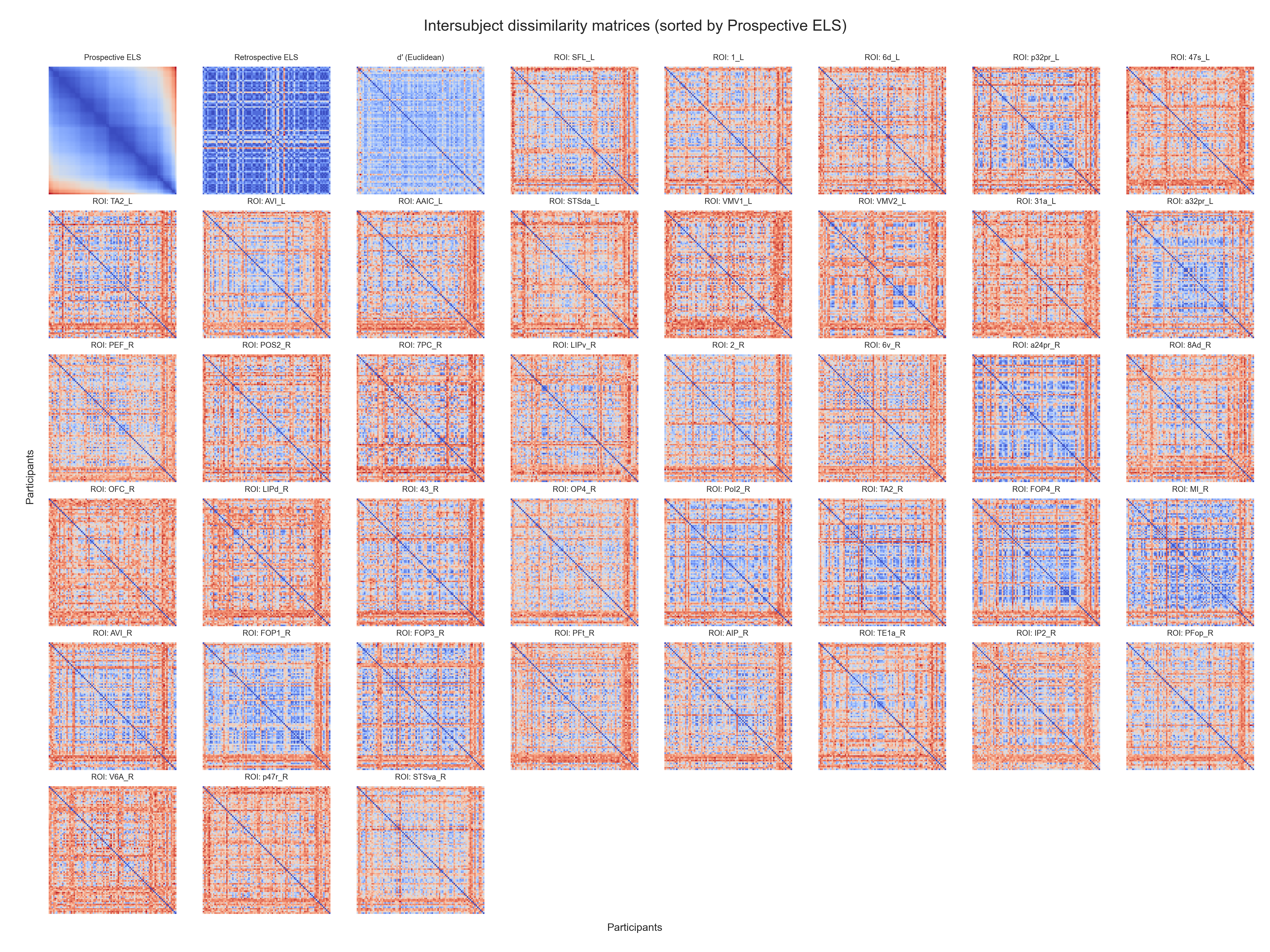

Supplement: Supplementary file 8 — FIGURE S8: Visualizations of ISDMs for Prospective ELS (overall score), Retrospective ELS, d‐prime, and the 40 regions with significant IS‐RSA correlations between pairwise distances in Prospective ELS and pairwise (correlation) distance in RDMs. ISDMs are sorted according to Prospective ELS. [file HBM-46-e70373-s003.png]

# Sorted correlations between ELS ISDMs and the 360 ROI ISDMs

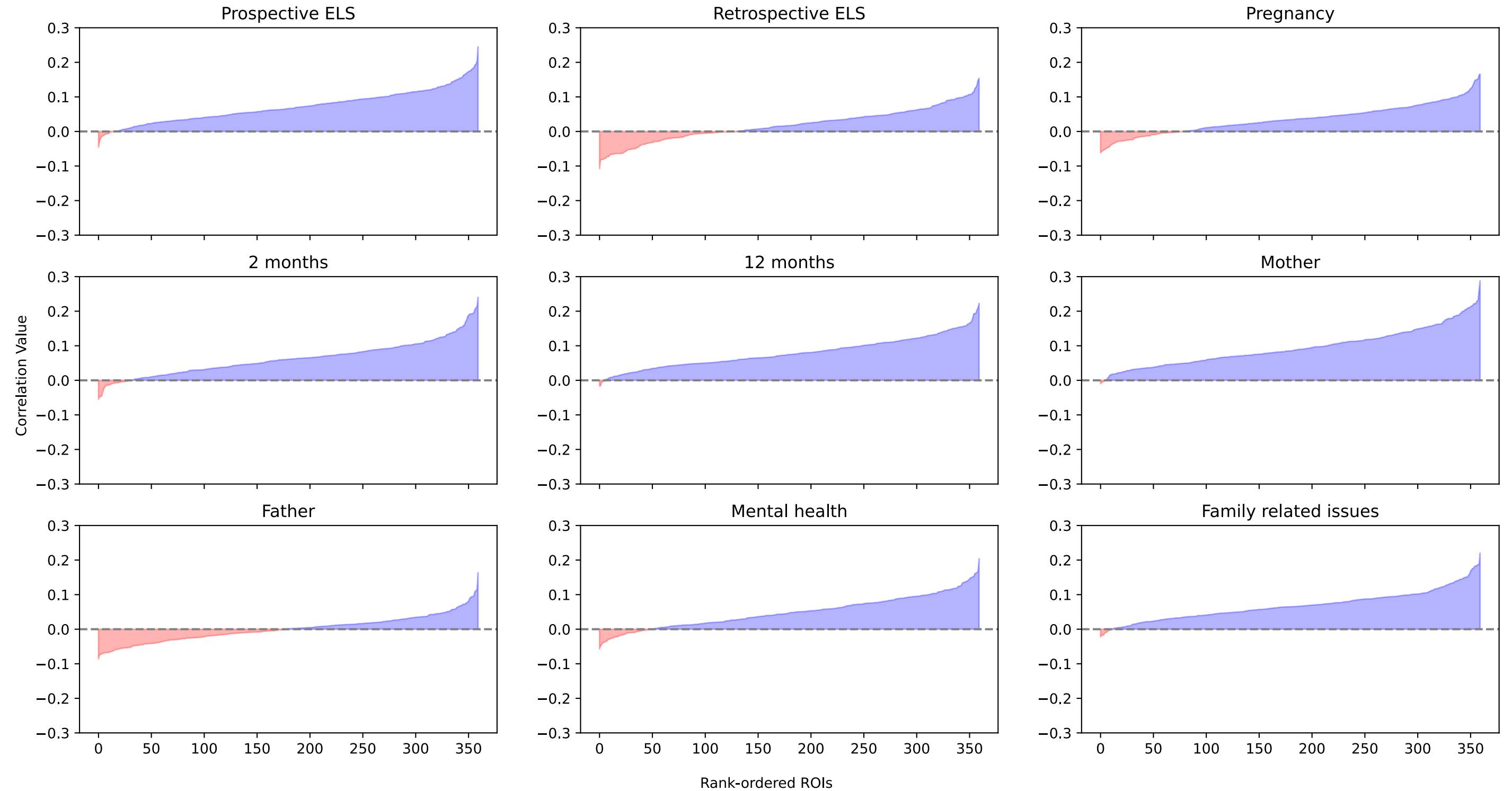

Supplement: Supplementary file 9 — FIGURE S9: Sorted correlation coefficients for intersubject representational similarity analysis correlations between different ELS measurements and the 360 ROI specific RDMs. Correlations have been sorted within each ELS measurement to demonstrate the distribution and ratio of negative and positive correlations. Correlations were attained from partial Spearman's correlation between the vectorized intersubject dissimilarity matrices constructed from various ELS measurements (Prospective and Retrospective ELS, and the 7 different domains of the Prospective ELS), and the 360 intersubject dissimilarity matrices constructed from pairwise correlation distances between vectorized representational dissimilarity matrices for each cortical region of the Human Connectome Project Multimodal Parcellation 1.0. Correlations depicted include: Prospective ELS (overall Prospective ELS score), Retrospective ELS (adapted revised Adverse Childhood Experiences questionnaire), Pregnancy (Prospective ELS parent reports in pregnancy over all questionnaires), 2 months (Prospective ELS parent reports when the child was 2 months old over all questionnaires), 12 months (Prospective ELS parent reports when the child was 12 months old over all questionnaires), Mother (mother's Prospective ELS reports over all timepoints and questionnaires), Father (father's prospective ELS reports over all timepoints and questionnaires), Mental health (BDI and GHQ from both parents over all timepoints), Family related issues (DAS and PSI from both parents over all timepoints). [file HBM-46-e70373-s001.pdf]
