# Supplementary material for: Similarity in Early Life Stress Exposure Is Associated With Similarity in Neural Representations in Early Adulthood
Source: Hum Brain Mapp. 2025 Oct 4;46(14):e70373. doi: 10.1002/hbm.70373 (PMC12495272; doi:10.1002/hbm.70373)
Supplement: Supplementary file 4 — FIGURE S4: Visualizations for the averages (mean) and standard deviations of each RDM element depicting Spearman's correlations between pairwise dissimilarities in the RDM element and pairwise dissimilarity in Prospective ELS. To attain the matrices, first, the RDMs' absolute element‐wise differences were calculated for each unique pair of participants within each ROI that had a significant IS‐RSA correlation (yielding difference‐RDMs). Then, within each ROI, a Spearman correlation was calculated between the difference‐RDM elements and pairwise dissimilarity in Prospective ELS, yielding a matrix whose elements denote the direction and consistency of change in that element's pairwise dissimilarities as a function of Prospective ELS dissimilarity. Finally, the mean and standard deviation values of each element of these matrices were calculated. The resulting matrices thus denote which elements of the difference‐RDM change most strongly and consistently as a function of pairwise dissimilarities in Prospective ELS, and how much variation there is in this strength and consistency within each of the four clusters. [file HBM-46-e70373-s007.pdf]

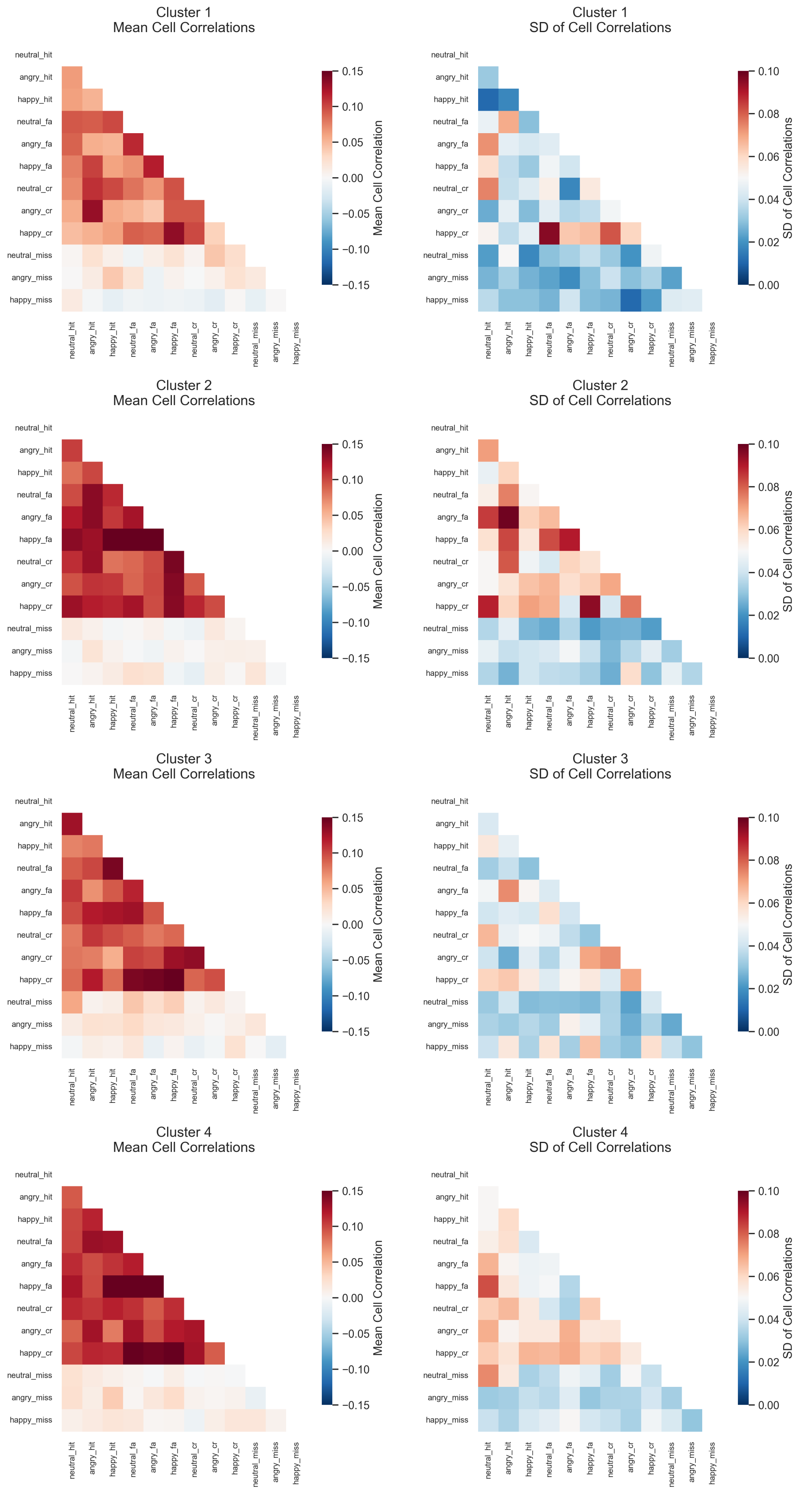

Cluster 1
Mean Cell Correlations
Cluster 1
SD of Cell Correlations
Cluster 2
Mean Cell Correlations
Cluster 2
SD of Cell Correlations
Cluster 3
Mean Cell Correlations
Cluster 3
SD of Cell Correlations
Cluster 4
Mean Cell Correlations
Cluster 4
SD of Cell Correlations
neutral_hit
angry_hit
happy_hit
neutral_fa
angry_fa
happy_fa
neutral_cr
angry_cr
happy_cr
neutral_miss
angry_miss
happy_miss
Mean Cell Correlation
0.15
0.10
0.05
0.00
−0.05
−0.10
−0.15
SD of Cell Correlations
0.10
0.08
0.06
0.04
0.02
0.00
